# Supramolecular assemblies of amphiphilic donor–acceptor Stenhouse adducts as macroscopic soft scaffolds

**DOI:** 10.3762/bjoc.20.142

**Published:** 2024-07-15

**Authors:** Ka-Lung Hung, Leong-Hung Cheung, Yikun Ren, Ming-Hin Chau, Yan-Yi Lam, Takashi Kajitani, Franco King-Chi Leung

**Affiliations:** 1 The Hong Kong Polytechnic University Shenzhen Research Institute, Shenzhen 518057, China; 2 State Key Laboratory of Chemical Biology and Drug Discovery, Department of Applied Biology and Chemical Technology, The Hong Kong Polytechnic University, Hong Kong, China; 3 Open Facility Development Office, Open Facility Center, Tokyo Institute of Technology, 4259 Nagatsuta, Midori-ku, Yokohama 226-8503, Japan; 4 Centre for Eye and Vision Research, 17W Hong Kong Science Park, Hong Kong, China

**Keywords:** donor–acceptor Stenhouse adduct, photoresponsive molecular amphiphile, supramolecular transformation, visible light

## Abstract

In the design of photoharvesting and photoresponsive supramolecular systems in aqueous medium, the fabrication of amphiphilic photoswitches enables a noninvasive functional response through photoirradiation. Although most aqueous supramolecular assemblies are driven by high-energy and biodamaging UV light, we have previously reported a design of amphiphilic donor–acceptor Stenhouse adducts (DASAs) controlled by white light. Herein, we present a series of DASA amphiphiles (DAs) with minor structural modifications on the alkyl linker chain length connecting the DASA motif with the hydrophilic moiety. The excellent photoswitchability in organic medium and the photoresponsiveness in aqueous medium, driven by visible light, were investigated by UV–vis absorption spectroscopy. The assembled supramolecular nanostructures were confirmed by electron microscopy, while the supramolecular packing was revealed by X-ray diffraction analysis. Upon visible-light irradiation, significant transformations of the DA geometry enabled transformations of the supramolecular assemblies on a microscopic scale, subsequently disassembling macroscopic soft scaffolds of DAs. The current work shows promising use for the fabrication of visible-light-controlled macroscopic scaffolds, offering the next generation of biomedical materials with visible-light-controlled microenvironments and future soft-robotic systems.

## Introduction

Solar energy is of paramount importance to life on the earth for various reasons, such as maintenance of a stable temperature and enabling photosynthesis as the basis of the food chain. Inspired by the natural photosynthetic processes, synthetic molecules were designed and functionalized with photoresponsive and photoabsorbing functional motifs, serving as the counterpart of natural photosystems. This allowed to construct smart materials that harvest light energy, e.g., solar cells, photosensitizers, and photochromic materials [1–4]. Supramolecular assemblies are commonly found in nature in order to enable different biological functions in a precise manner, e.g., actin in biological molecular motors, the cytoskeleton, and cell membrane [5–8]. Mimicking natural supramolecular assemblies in aqueous medium, the intrinsic supramolecular dynamicity, stimuli-responsiveness, and molecular functional tunability of synthetic supramolecular systems can be precisely controlled through the delicate design and synthesis of organic molecules [9–14]. Synthetic supramolecular systems can respond to various external stimuli, e.g., light, pH, organic solvents, ions, and heat [15–22]. Light enables noninvasive stimulation with high spatial and temporal precision to control structural transformations of supramolecular assemblies in organic and aqueous media [19,23–25]. Various photochromic and photoresponsive moieties, such as, stiff-stilbene [26], azobenzene [27–28], molecular motors [19,29–30], spiropyran [31–33], indigo [34–35], and donor–acceptor Stenhouse adducts (DASAs) [36–37], have been used in supramolecular systems for photoswitchable smart electronic, optoelectronic, and biomedical materials [30,38–40].

Photoresponsive supramolecular amphiphiles are responsive, complex, and adaptable in aqueous medium [41–43]. Supramolecular assemblies of photoresponsive molecular amphiphiles in aqueous medium can undergo transformation in solution or at the air–water interface and even sustained artificial muscle-like functions [17,43–44]. To tackle poor biocompatibility observed in UV-driven supramolecular assemblies of photoresponsive molecular amphiphiles, visible-light-active supramolecular systems enabling transformations across multiple length scales are urgently required. Some visible-light-driven supramolecular assemblies on a microscopic length scale, such as those containing spiropyrans, azobenzenes, and indigos, have been used in transformations in aqueous medium [35,45–46]. However, very few examples of visible-light-driven supramolecular transformation on a macroscopic length scale are available. UV-light-driven supramolecular transformations on a macroscopic length scale were pioneered by Leung and Feringa [42–43]. Besides, we recently reported the first indigo amphiphiles in fabrication of macroscopic soft scaffolds with photoshrinking properties as a smart functional cell-material interfaces [34].

**Scheme 1:**
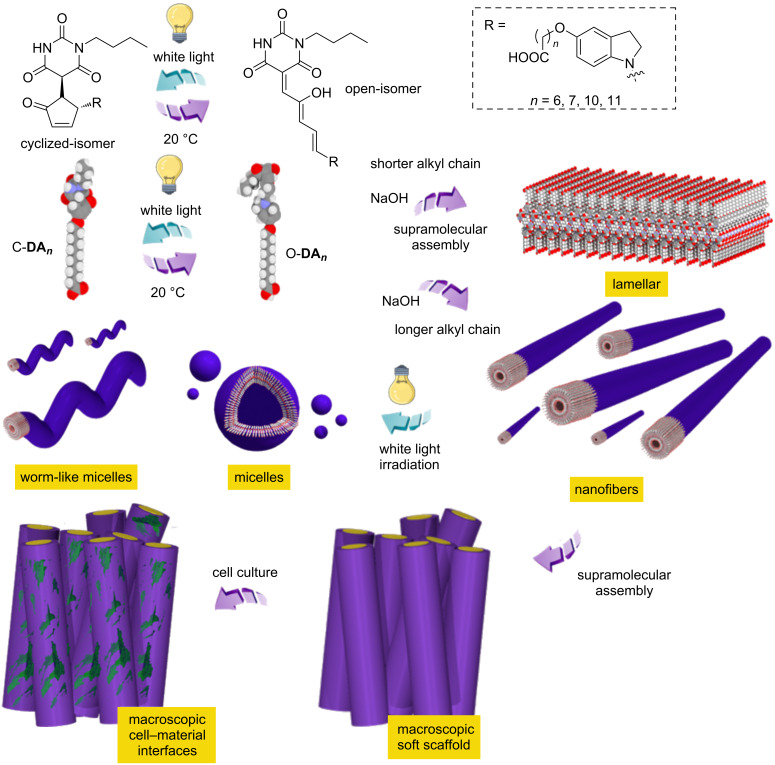
Illustration of the reversible visible-light-controlled ring closure and thermal-driven ring-opening-processes of **DA*_n_*** and supramolecular assemblies on different length scales.

Alternatively, the visible-light-responsiveness of DASAs was firstly reported by Read de Alaniz [47–48]. The significant structural changes upon transformation of the open-isomer to the cyclized-isomer, and the simple modular synthesis of DASAs, enabled various applications in photochromic systems and smart functional materials [47,49–59]. Inspired by the molecular designs of DASAs, we developed the first DASA amphiphiles (DAs) assembled into large-aspect-ratio nanostructures in aqueous medium while sustaining the visible-light-controlled supramolecular structural transformations [37]. The large-aspect-ratio DA nanostructures assembled into macroscopic soft scaffolds, for which the disassembly process on a macroscopic length scale was controlled by white light. Furthermore, the red-light-responsiveness of the DAs could be regained in aqueous medium upon coassembly with stiff-stilbene amphiphile due to reduced intermolecular stacking [36]. The excellent photoresponsiveness of DAs across multiple length scales significantly increases the urgency to investigate the biocompatibility and molecular structural derivatives. Based on the molecular design of the reported DAs, we designed and synthesized a series of DAs featuring a second-generation DASA switching motif and different chain lengths of the alkyl linker (i.e., **DA*_n_***) that connects the DASA indoline motif with the hydrophilic part (i.e., the carboxylic acid motif, Scheme 1). The alkyl linker length allowed fine adjustment of the hydrophobic volume of **DA*_n_***, enabling significant packing parameters changes upon supramolecular assembly in aqueous medium. On a microscopic length scale, the expected supramolecular assembly and the related assembly transformations can be controlled systematically by visible light irradiation. On a macroscopic length scale, the soft **DA*_n_*** scaffolds were able to perform macroscopic structure disassembly upon visible-light irradiation. The biocompatibility of the macroscopic soft **DA*_n_*** scaffolds was investigated, revealing limited cytotoxicity. The current work could open up new prospects in the development of biomedical materials with visible-light-controlled microenvironments and future soft robotic systems.

## Results and Discussion

### Synthesis and design of DAs

**Scheme 2:**
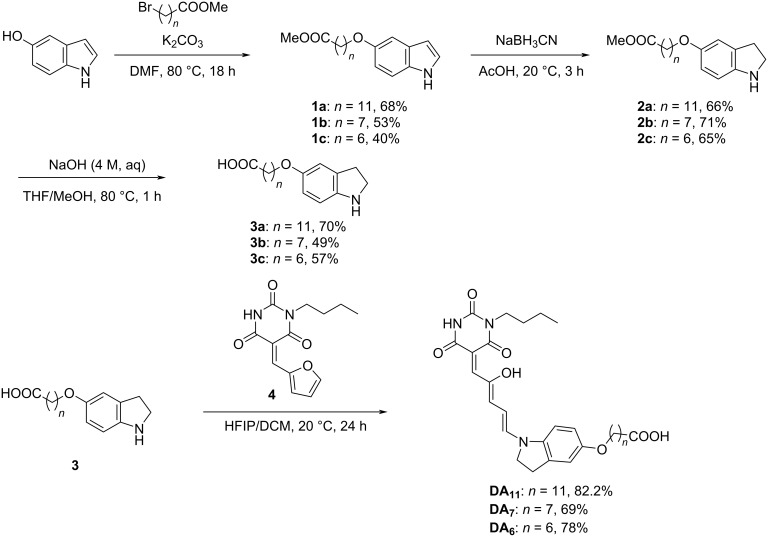
Synthetic pathway to **DA*_n_***.

We designed and synthesized a series of **DA*_n_*** with different chain lengths of the alkyl linker, i.e., number of methylene groups in the alkyl linker (*n*) = 11 (in **DA_11_**), 7 (in **DA_7_**), and 6 (in **DA_6_**). The effect of the alkyl linker length in **DA_11_**, **DA_7_**, and **DA_6_** is discussed and compared to our previously reported **DA_10_** [37]. The donor part of **DA*_n_*** was synthesized by alkylation in the presence of K_2_CO_3_ in a nitrogen atmosphere, followed by reduction of the indole motif in compound **1*_n_*** to indoline in **2*_n_***. The ester group in compound **2*_n_*** was deprotected under basic conditions to give compound **3*_n_*** (Scheme 2). **DA*_n_*** with different chain lengths of the alkyl linker were synthesized through an aza-Piancatalli rearrangement between a barbiturate–furan adduct **4** and compound **3*_n_*** under ambient conditions in dichloromethane and hexafluoro-2-propanol (HFIP). The synthetic procedures and characterization of all newly synthesized compounds, including **DA_11_**, **DA_7_**, and **DA_6_**, are summarized in Supporting Information File 1, Figures S13–S33.

### Photochemical properties of **DA*_n_*** in organic medium

**Figure 1:**
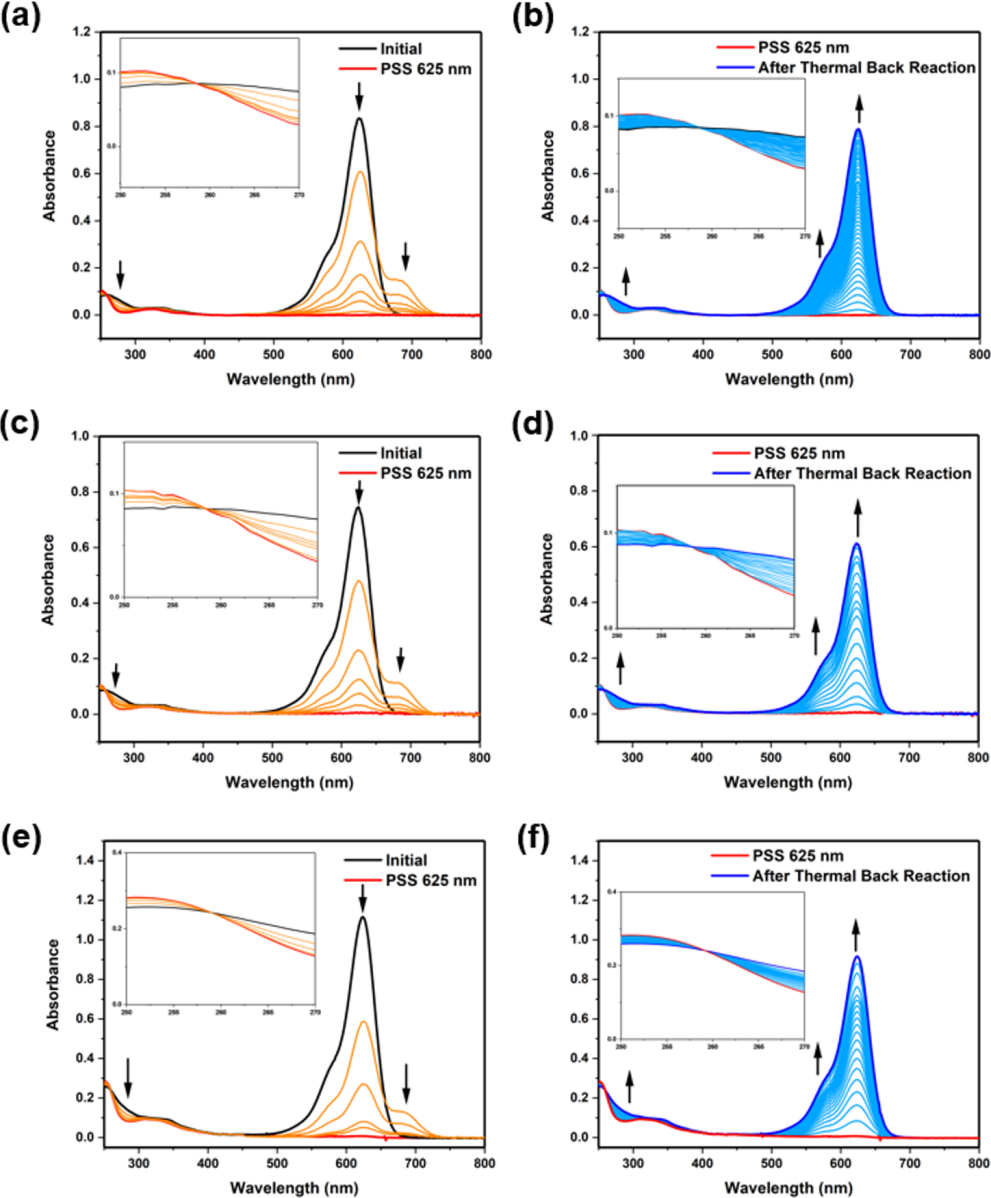
UV–vis-absorption-spectral changes of **DA*_n_*** in THF solution (20 μM). (a) **DA_11_**, (c) **DA_7_**, (e) **DA_6_** solutions in the beginning (black line), upon 625-nm irradiation over the course of 1 min (orange lines), and after irradiation to reach the PSS (red line). (b) **DA_11_**, (d) **DA_7_**, (f) **DA_6_** upon thermal back reaction over the course of 60 min at 20 °C (cyan lines) and after thermal back reaction (blue line). Insets: enlarged 250–270 nm range. Irradiation was carried out using a Thorlabs model M625F2 high-power LED (625 nm, 1.0 A) positioned at a distance of 1 cm from the sample.

The photochemical properties of **DA*_n_*** were first studied in organic solvent by UV–vis absorption spectroscopy. The synthesized **DA_11_** in THF solution (20 µM) showed a strong absorption band at 470–685 nm in the UV–vis absorption spectrum (Figure 1a). Upon 625 nm red-light irradiation for 60 s at 20 °C to reach the photostationary state (PSS), the strong absorption band at 470–685 nm was diminished, with a clear isosbestic point at 259 nm (Figure 1a, red line), which indicates a selective photoisomerization process from the open-isomer O-**DA_11_** (Figure 1a, black line and Figure 1b blue line) to the cyclized-isomer C-**DA_11_** (Figure 1a, red line). The resulting solution continued to be irradiate with 625 nm red light for 60 s at 20 °C and subsequently stored in the dark at 20 °C for 60 min for the thermal back reaction to occur to test the reversibility and photostability in organic solvent (Figure S1a and S1b, Supporting Information File 1). The photoisomerization between O-**DA_11_** and C-**DA_11_**, upon irradiation with 625 nm red light and thermal back reaction in the dark, could be repeated for over five cycles, with only 5% absorbance decreases per cycle (Figure S1a and S1b, Supporting Information File 1). In contrast, the previously studied reversibility of the photoswitching of **DA_10_** had revealed 10% absorbance decreases per cycle [37], indicating a mild improvement to the fatigue resistance of **DA_11_**.

To further investigate the photostability and reversibility of the other compounds, **DA*_n_*** with shorter alkyl linkers were also examined in THF solvent by UV–vis absorption spectroscopy, where they showed photochemical properties similar to those of **DA_11_** (Figure 1a and Figure 1b). THF solutions of **DA_7_** and **DA_6_** showed a strong absorption band at 470–685 nm (Figure 1c and Figure 1e). Upon red-light irradiation at 625 nm for 60 s at 20 °C to reach the PSS, the strong absorption band was diminished, similarly to **DA_11_**, with a clear isosbestic point at 259 nm (Figure 1c and Figure 1e, red line). After thermal back reaction in the dark at 20 °C for 60 min (Figure 1d and Figure 1f, blue line), the strong absorption band had recovered and indicated a selective photoisomerization process from the open-isomers O-**DA_7_** and O-**DA_6_** (Figure 1c–f, black and blue lines) to the cyclized-isomers C-**DA_7_** and C-**DA_6_** (Figure 1d and Figure 1f, red line). The results showed feasible photoreversibility of **DA_7_** and **DA_6_** for five additional photoswitching cycles. However, a more significant fatigue effect was observed in **DA_7_** (Figure S1c and S1d, Supporting Information File 1) and **DA_6_** (Figure S1e and S1f, Supporting Information File 1), with 15% absorbance decline per cycle. As a stronger fatigue effect was observed in the photoisomerization of **DA_7_** and **DA_6_**, this might be attributed to stabilization through hydrogen bonding and a favored cyclized-isomer in polar solvents. The increased alkyl linker length of **DA_10_** and **DA_11_** provided an improved photostability compared to **DA_7_** and **DA_6_**, possibly due to the reduced stabilization and improved fatigue resistance of the cyclized-isomer in a polar environment.

### Photochemical properties of **DA*_n_*** in aqueous medium

**Figure 2:**
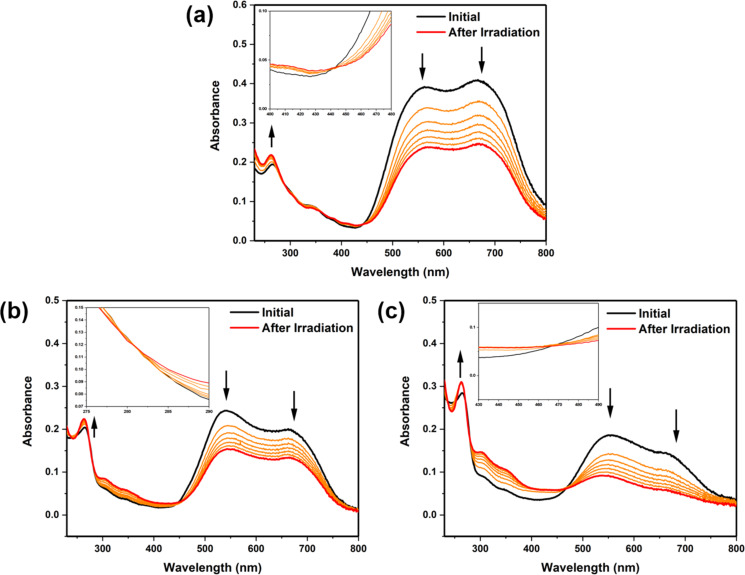
UV–vis absorption spectra of **DA*_n_*** in aqueous solution (43 μM). (a) **DA_11_** (inset: enlarged 400–480 nm area), (b) **DA_7_** (inset: enlarged 275–290 nm area), (c) **DA_6_** (inset: enlarged 430–490 nm area) in the beginning (black line), upon white-light irradiation over the course of 60 min (orange lines), and after 60 min (red line). Irradiation was carried out at 20 °C using a light-guide-equipped BBZM-I xenon light source (380–800 nm, 300 W) positioned at a distance of 1 cm from the sample.

Delighted by the good photoswitchability performance of **DA*_n_*** in organic solvent, the photochemical properties and supramolecular assembly were investigated in aqueous medium. An aqueous solution of 5.0 wt % **DA_11_** in Milli-Q water was prepared, followed by deprotonation with 1.0 equivalent of NaOH, showing excellent solubility up to 93 mM, giving a deep purple solution. An aqueous 43 µM solution **DA_11_**, diluted from the 5.0 wt % stock solution, was investigated by UV–vis absorption spectroscopy. Absorption bands at 250–325 nm and 430–800 nm were observed (Figure 2a), wherein the maximum at 667 nm was accompanied by a shoulder at 561 nm, similarly to the spectrum of **DA_10_** [37]. A significant spectral shift and peak broadening of **DA_11_** were observed compared to the THF solution (Figure 1a, black line), possibly due to supramolecular assembly in aqueous medium. Because of the broad absorption range of **DA_11_** in aqueous medium, photoisomerization could not be effectively accomplished by a 625 nm LED light source with a narrow emission wavelength range, similarly to our previously studies [37]. Thus, a white-light source with a broad emission wavelength range was employed for the photoisomerization of **DA*_n_*** in aqueous medium. Photoisomerization of **DA_11_** was induced by white-light irradiation for 60 min at 20 °C (Figure 2a, orange line), upon which the absorption band at 430–800 nm decreased and that at 250–325 nm increased. At the same time, isosbestic points were clearly observed at 286 nm, 357 nm, and 443 nm (Figure 2a, red line). The results indicated a slower selective photoisomerization process from O-**DA_11_** to C-**DA_11_** in aqueous medium compared to the THF (Figure 1a). The thermal back reaction in the resulting aqueous solution was further studied, showing no reverse switching to O-**DA_11_** upon storage in the dark at 20 °C (Figure S2, Supporting Information File 1). It has previously been observed that the cyclized-isomers of common DASAs remained stable in aqueous medium, rendering the photoisomerization process irreversible [47–48,60]. The hindered thermal back reaction of **DA_11_** should also be attributed to a phenomenon similar to that observed in our previously reported **DA_10_** [37].

The **DA*_n_*** with a shorter alkyl linker, i.e., **DA_7_** and **DA_6_**, in aqueous solution were also investigated in a protocol identical to that outlined above. An aqueous 43 µM solution of **DA_7_** had absorption bands at 250–325 nm and 430–800 nm, with a maximum at 543 nm, accompanied by a shoulder at 667 nm (Figure 2b). An aqueous 43 µM solution of **DA_6_** similarly had absorption bands at 250–325 nm and 430–800 nm, a broad absorption peak at 557 nm with a shoulder at 667 nm, and a maximum at 263 nm (Figure 2c). Upon white-light irradiation for 60 min at 20 °C, the absorption band at 430–800 nm was decreased, which could be observed in both **DA_7_** (Figure 2b, orange line) and **DA_6_** (Figure 2c, orange line), whereas the absorption band at 250–470 nm was increased. After irradiation for 60 min at 20 °C, distinct isosbestic points were located at 286 nm and 449 nm for **DA_7_** (Figure 2b, red line,) and at 467 nm and 283 nm for **DA_6_** (Figure 2c, red line,), indicating selective photoisomerization from O-**DA_7_** to C-**DA_7_** and from O-**DA_6_** to C-**DA_6_** in aqueous solutions. No thermal back reaction was observed for **DA_7_** (Figure S3, Supporting Information File 1) and **DA_6_** (Figure S4, Supporting Information File 1), similarly to **DA_11_** and **DA_10_**. The results indicated that even a subtle modification of the alkyl linker length could induce an obvious spectral shift, implying different supramolecular assemblies of **DA*_n_*** in aqueous medium.

### Photocontrolled supramolecular assemblies of **DA*_n_*** in aqueous medium

**Figure 3:**
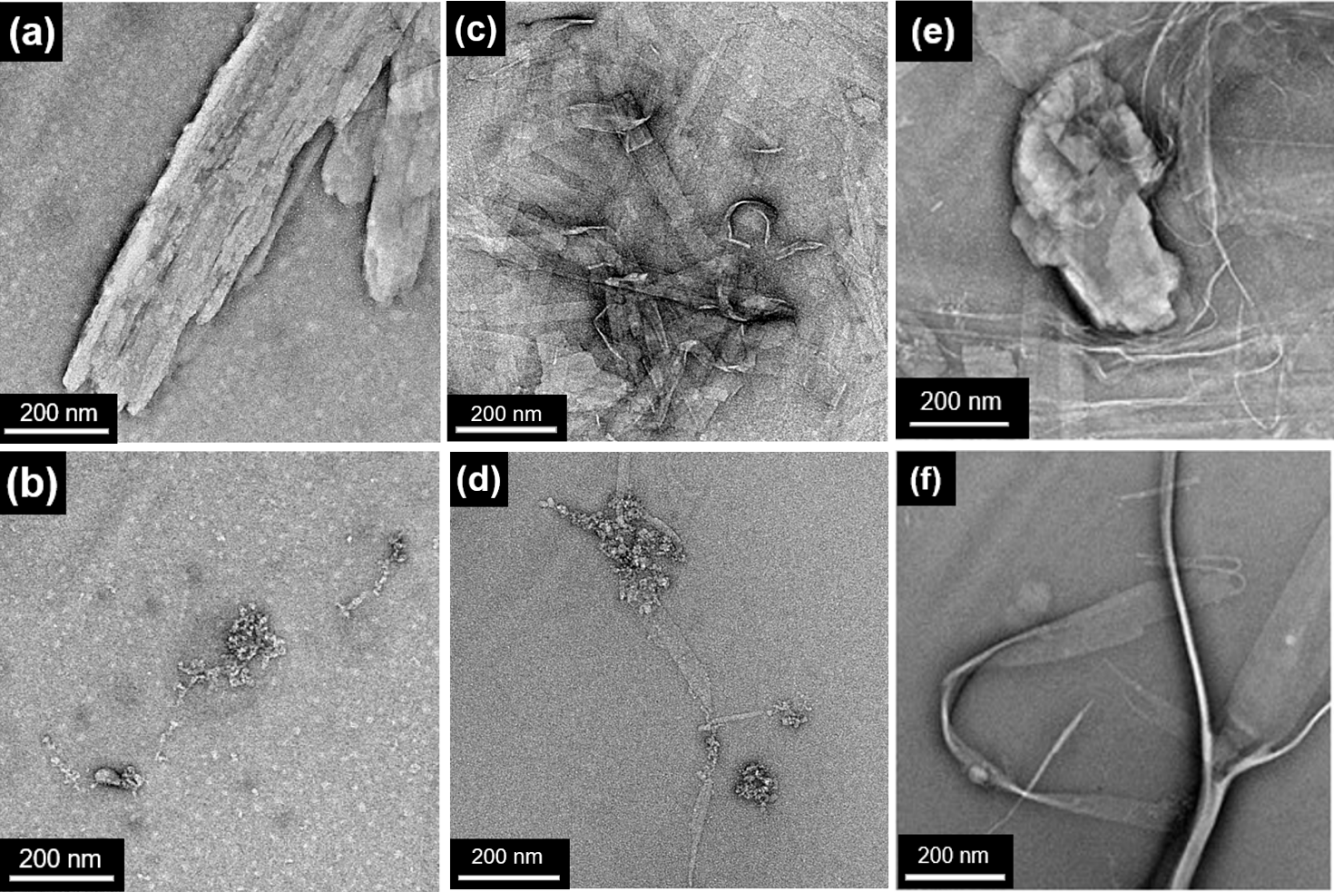
TEM images of freshly prepared aqueous solutions before irradiation of (a) **DA_11_** (0.25 wt %, 4.1 mM), (c) **DA_7_** (0.25 wt %, 4.5 mM), and (e) **DA_6_** (0.25 wt %, 4.6 mM). The solutions of (b) **DA_11_**, (d) **DA_7_**, and (f) **DA_6_** were irradiated with white light for 60 min at 20 °C.

A freshly prepared aqueous solution of **DA_11_** (4.1 mM, 0.25 wt %), in the presence of 1.0 equivalent of NaOH, was further diluted to prepare various aliquots with a concentration ranging from 1.0 × 10^–4^–0.1 mM in order to estimate the critical aggregation concentration (CAC) by using static light scattering (SLS). An aqueous solution of **DA_11_** was estimated to have a CAC of <6.0 μM (Figure S5, Supporting Information File 1). Negative-stain transmission electron microscopy (TEM) was employed to investigate the supramolecular transformations in aqueous medium. An aqueous solution of **DA_11_** (82 mM, 5.0 wt %) was diluted to 4.1 mM (0.25 wt %), revealing large-aspect-ratio supramolecular nanofiber assemblies bundled ≈100 nm in width and hundreds of nm in length (Figure 3a and Figure S8a, Supporting Information File 1). Upon white-light irradiation for 60 min at 20 °C, the photoisomerization of **DA_11_** at 4.1 mM transformed the supramolecular assembly from bundled nanofibers into a mixture of worm-like micelles and micelle structures 3–4 nm in diameter (Figure 3b and Figure S8b, Supporting Information File 1). The supramolecular structure of **DA_11_** and the supramolecular transformation were similar to those observed in **DA_10_** [37]. This subtle increase in alkyl linker length in **DA_11_** gave an essentially identical performance compared to **DA_10_** on a microscopic length scale. Using the same protocol, aqueous solutions of **DA_7_** (0.25 wt %, 4.5 mM) and **DA_6_** (0.25 wt %, 4.6 mM) were prepared by dilution of a 5.0 wt % solution in the presence of 1.0 equivalent of NaOH. The CAC of aqueous solutions of **DA_7_** and **DA_6_** was estimated to be <0.05 mM (Figure S6, Supporting Information File 1) and <0.1 mM (Figure S7, Supporting Information File 1), respectively. This suggested that a longer **DA*_n_*** alkyl chain led to a lower CAC due to the higher propensity for supramolecular assembly into nanostructures. In aqueous solution, **DA_7_** (0.25 wt %) formed lamellar structures ≈200 nm in length and ≈40 nm in width (Figure 3c and Figure S8c, Supporting Information File 1), as observed by TEM. A similar lamellar structure was found in an aqueous solution of **DA_6_** (Figure 3e and Figure S8e, Supporting Information File 1). More significant structural variations were revealed upon decreasing the alkyl linker length from *n* = 11 to *n* = 7 or 6. Similarly, the lamellar structure of **DA_7_** disassembled into worm-like micellar structures upon white-light irradiation (Figure 3d and Figure S8d, Supporting Information File 1), while the lamellar structure of **DA_6_** remained unchanged (Figure 3f and Figure S8f, Supporting Information File 1), possibly due to the stability of the resulting supramolecular structures. The photoswitchability and structural transformations of the supramolecular assemblies on a microscopic length scale opened up possibilities for **DA*_n_*** to be used for the fabrication of macroscopic soft scaffolds after charge screening with complementary ions.

### Fabrication and characterization of macroscopic soft scaffolds of **DA*_n_*** in aqueous medium

**Figure 4:**
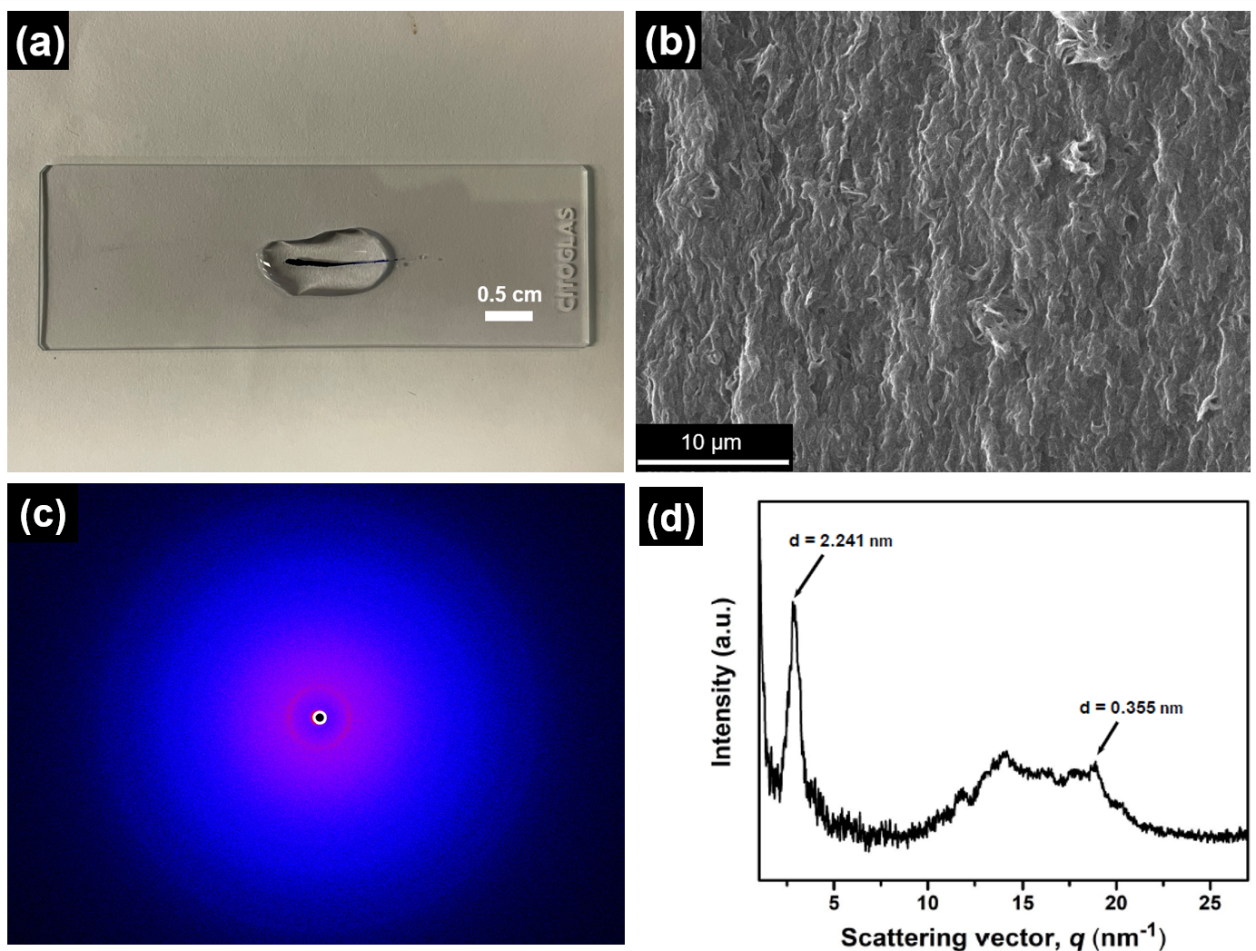
Photograph of a freshly prepared aqueous **DA_11_** solution (82.0 mM) ejected into a shallow pool of CaCl_2_ solution (150 mM), (b) SEM image of a macroscopic **DA_11_** scaffold prepared from a solution of CaCl_2_ (150 mM), (c) 2D WAXD image of **DA_11_** macroscopic soft scaffold with an X-ray exposure time of 20 min, and (d) 1D WAXD pattern of a 2D WAXD image of a **DA_11_** macroscopic soft scaffold in (c).

A freshly prepared aqueous solution of **DA_11_** (5.0 wt %, 82 mM) was deep blue and remained stable for months. By applying the shear-flow method, negatively charged nanofibers of **DA_11_** were assembled into a macroscopic soft scaffold when the solution was ejected into a shallow pool of calcium chloride solution (150 mM, Figure 4a). The high binding affinity between calcium ions and carboxylate groups enabled charge screening of **DA_11_** and stabilization as macroscopic soft scaffold. Under an optical microscope, a deep-blue string with a diameter of ≈560 μm was observed in the macroscopic soft scaffold of **DA_11_** (Figure S9, Supporting Information File 1), without any birefringence under crossed polarizers. A macroscopic soft scaffold of **DA_11_** was imaged by scanning electron microscopy (SEM) to reveal bundled nanofibers partially aligned with the long axis of the macroscopic soft scaffold (Figure 4b). The degree of alignment (structural parameters and orientational order) of macroscopic soft scaffolds of **DA_11_** were investigated with through-view wide-angle X-ray diffraction (WAXD). The 2D WAXD image of the macroscopic soft scaffold of **DA_11_** showed a diffraction ring in the region of *q* = 0.5–5.0 nm^−1^ (Figure 4c), which was similar to that of **DA_10_** [37]. A low degree of angular dependency was observed in the macroscopic soft scaffold of **DA_11_**, with a pair of weak peak maxima with full-width half-maxima at ≈110° (Figure S10, Supporting Information File 1), while the **DA_10_** macroscopic soft scaffold showed no obvious peak maximum. The results indicated subtle unidirectional alignment of bundled **DA_11_** with respect to the long axis of the macroscopic soft scaffold, while no significant unidirectional alignment was observed in the **DA_10_** macroscopic soft scaffold. The 1D WAXD pattern of the 2D WAXD image of the **DA_11_** macroscopic soft scaffold showed diffraction peaks at *d* = 2.24 nm and 0.36 nm (Figure 4d). The diffraction peak at *d* = 2.24 nm could be attributed to the longer molecular axis of **DA_11_** in dimer form, while that at *d* = 0.36 nm originated from π–π stacking of the DASA motif. Consistently, diffraction from the longer molecular axis of **DA_10_** in dimer form was shorter (*d* = 2.19 nm) than that of **DA_11_**, revealing the subtle alkyl linker length variation of **DA*_n_*** even on a macroscopic length scale. Further verifying this variation in the packing structure, the 2D WAXD images of **DA_7_** and **DA_6_** macroscopic soft scaffolds (Figures S11a and S12a, Supporting Information File 1, respectively) showed diffraction rings with *d*-spacings at 1.50 nm and 1.45 nm, respectively (Figure S11b and Figure S12b, Supporting Information File 1), revealing the shortened longer molecular axis of **DA_7_** and **DA_6_** in dimer form compared to **DA_11_** and **DA_10_**. The observed major diffraction peaks of **DA_7_** and **DA_6_** macroscopic soft scaffolds also originated from π–π stacking of the DASA motifs. However, no significant unidirectional alignment was observed in WAXD and SEM images of **DA_7_** and **DA_6_** macroscopic soft scaffolds (Figure S11a and S11c as well as S12a and S12c, Supporting Information File 1, respectively). Electron microscopy and WAXD revealed that a longer alkyl linker length of **DA*_n_*** could slightly improve the degree of unidirectional alignment of **DA*_n_*** nanostructures in the corresponding macroscopic soft scaffolds, possibly due to the higher tendency to bundle.

### Photocontrolled disassembly and biocompatibility of macroscopic soft scaffolds of **DA*_n_***

**Figure 5:**
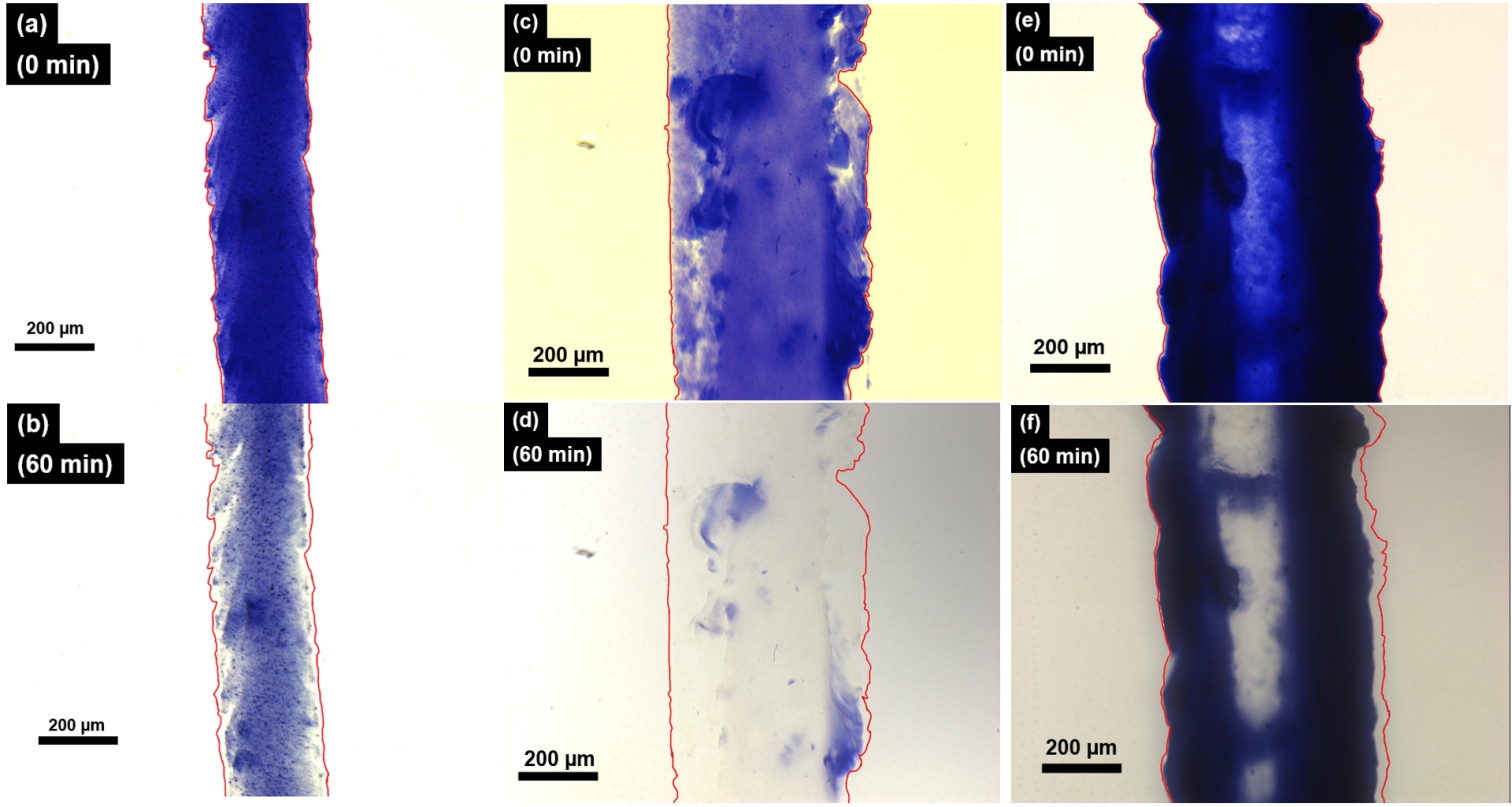
Photographs of macroscopic soft scaffolds prepared from aqueous solutions of (a) **DA_11_** (32.9 mM), (c) **DA_7_** (36.2 mM), (e) **DA_6_** (37.2 mM) in the presence of CaCl_2_ (150 mM) before white-light irradiation and (b) **DA_11_**, (d) **DA_7_**, and (f) **DA_6_** after white-light irradiation for 60 min.

Macroscopic soft scaffolds of **DA*_n_*** were shown to have an excellent structural stability and to undergo supramolecular transformations through visible light. A macroscopic **DA_11_** soft scaffold was monitored over the course of visible-light irradiation under an optical microscope. At the start, a deep-blue string with a diameter of ≈240 μm was observed (Figure 5a and Supporting Information File 2). The macroscopic soft scaffold weakened and shrank in the process. It also partially disassembled upon irradiation for 60 min (Figure 5b), consistent with the behavior of the **DA_10_** macroscopic soft scaffold. With the same treatment, the macroscopic soft scaffolds of **DA_7_** and **DA_6_** showed deep-blue strings with diameters of ≈500 μm and ≈570 μm, respectively (Figure 5c and Figure 5e as well as Supporting Information File 3 and Supporting Information File 4). After 60 min of white-light irradiation, the **DA_7_** and **DA_6_** scaffolds had significantly disassembled, and the deep-blue color had significantly faded as well (Figure 5d and Figure 5f). The results indicated that **DA*_n_*** macroscopic soft scaffolds enabled a photocontrolled disassembly process across multiple length scales.

**Figure 6:**
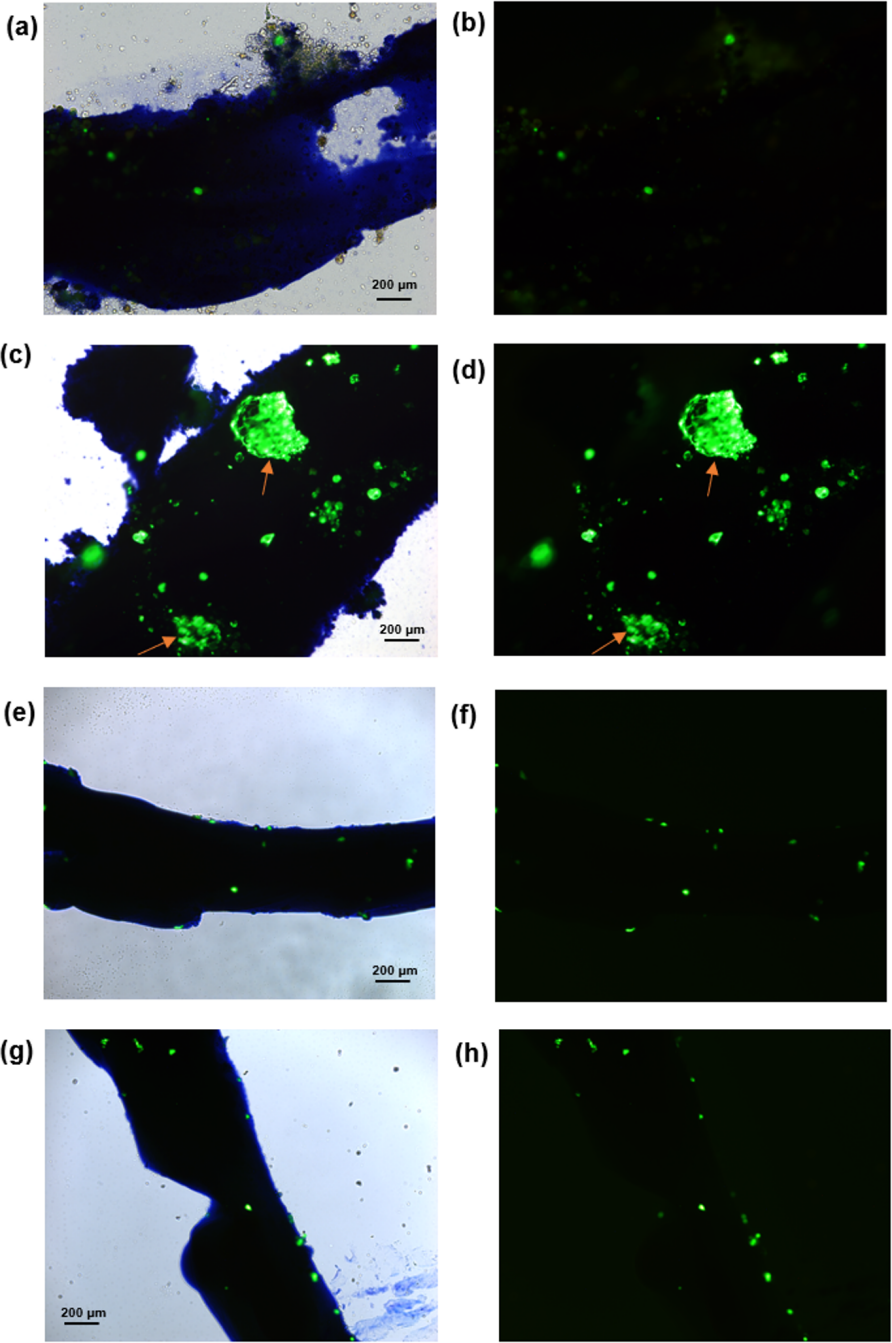
Macroscopic soft **DA*_n_*** scaffolds fabricated by the shear-flow method. Images taken during fluorescence microscopy. Photographs of freshly prepared (a) **DA_11_**, (c) **DA_10_**, (e) **DA_7_**, and (g) **DA_6_**, merged image of bright field and fluorescence with hBM-MSCs after 3 days of incubation. Fluorescence images of live cells in (b) **DA_11_**, (d) **DA_10_**, (f) **DA_7_**, and (h) **DA_6_** after 3 days of incubation to observe the cell affinity on the hydrogel surface. Scale bar: 200 μm, applied for all panels. The large fluorescent particles pointed at by orange arrows in (c) and (d) are the clustered hBM-MSCs.

Macroscopic soft **DA*_n_*** scaffolds were freshly prepared by ejecting 5.0 wt % **DA*_n_*** solutions onto a bioinert glass-bottomed Petri dish by the shear-flow method, rinsing with PBS, and incubating with human-bone-marrow-derived mesenchymal stem cells (hBM-MSCs) at 37 °C and 5% CO_2_ for 3 days. hBM-MSCs have demonstrated importance in controlled differentiations and clinical translations. The **DA_11_** scaffold remained intact after incubation at 37 °C, while the hBM-MSCs cells grew and attached onto the scaffold surface (Figure 6a and Figure 6b). However, the structural properties of the macroscopic soft **DA_11_** scaffold could not be transferred to the attached cells. Besides, the **DA_10_**, **DA_7_**, and **DA_6_** scaffolds, prepared in the same way, also showed intact macroscopic structures for 3 days of incubation at 37 °C, and hBM-MSCs cells attached onto the scaffold surfaces (Figure 6c–h). Though coverage of the **DA*_n_*** scaffolds surface with hBM-MSCs cells was limited, the cellular compatibility was good. These **DA*_n_*** biocompatibility results are the first example showing the intrinsic cytocompatibility of macroscopic soft DA scaffolds.

## Conclusion

Amphiphilic DASAs with alkyl linkers of different length connecting to a carboxylic acid were prepared. Photochemical properties of the **DA*_n_*** were investigated in both organic and aqueous media, revealing good photoswitchability in organic medium, while the thermal back reaction was hindered in aqueous medium. The large aspect ratio of the supramolecular **DA*_n_*** nanofibers in aqueous medium enabled supramolecular transformations upon white-light irradiation. The good stability of the nanostructures further allowed fabrication of macroscopic soft scaffolds with limited unidirectional alignment. The obtained **DA*_n_*** scaffolds showed photoresponsiveness in the form of partial weakening and disassembly after 60 min of irradiation. The scaffolds were further incubated with hBM-MSCs cells, promoting the attachment of cells onto the surface. The limited cytotoxicity and good stability of **DA*_n_*** macroscopic soft scaffolds points at an application as cell–material interface and even offers opportunities for the fabrication of biomedical materials with visible-light-controlled microenvironments and future soft robotic systems.

## Experimental

### Materials

All commercial reagents were purchased from Acros Organics, Aladdin, Alfa Aesar, Bidepharm, Dieckmann, Macklin, Sigma-Aldrich, and Tokyo Chemical Industry and used as received unless otherwise specified. All reactions were performed under nitrogen unless otherwise specified. Analytical thin-layer chromatography (TLC) was performed with Macherey-Nagel silica gel 60 UV254 aluminum plates, and visualization was accomplished by UV light (254 nm or 365 nm) or by staining with phosphomolybdic acid and heating. Flash column chromatography was performed using Macherey-Nagel silica gel 60 (230–400 mesh). Deuterated solvents were purchased from Cambridge Isotope Laboratories.

### UV–vis spectroscopy

UV–vis measurements were performed on an Agilent Cary 60 UV–vis spectrophotometer with a quartz cuvette of 1 cm path length. A Luma 40/Cary 60 temperature-controlled cuvette holder with four optical ports was mounted in the sample compartment. UV–vis irradiation was carried out at 20 °C using a Thorlabs model M625F2 high-power LED (625 nm, 1.0 A) (625 nm, 1.0 A) and a light-guide-equipped BBZM-I xenon light source (380–800 nm, 300 W) positioned at a distance of 1 cm from the sample.

### Preparation of aqueous samples

All aqueous solutions of **DA*_n_*** were prepared according to the following general procedure: **DA*_n_*** (5.0 wt %) was mixed with 1.0 equivalent NaOH in Milli-Q water. The obtained aqueous solution was sonicated for 10 min at room temperature to afford a deep-purple solution. The obtained solution was directly used or diluted for microscopic and spectroscopic measurements.

### TEM

TEM was performed on a JEOL model JEM-2010 transmission electron microscope with tungsten hair pin type filament operating at 120 kV, equipped with a Gatan 794 CCD camera. TEM samples were prepared by depositing sample solutions (5.0 μL) onto a carbon grid (micro to nano, EMR carbon support film on copper, 400 square mesh) for 20 s. The sample solution was removed by blotting, and UranyLess EM stain solution (5.0 μL, Electron Microscopy Science) was directly deposited onto the grid for 20 s, and the stain was removed by blotting.

### SEM and polarized optical microscopy analysis

Polarized optical microscopy was performed on a Leica DM2700-P optical polarizing microscope. SEM was performed on a Tescan VEGA3 scanning electron microscope. The preparation of soft scaffolds composed of **DA_6_**, **DA_7_**, and **DA_11_**, respectively, on a glass substrate was done as follows: Aqueous solutions of **DA_6_** (5.0 wt %, 92.9 mM), **DA_7_** (5.0 wt %, 90.5 mM), and **DA_11_** (5.0 wt %, 82.2 mM) were manually added to an aqueous solution of CaCl_2_ (150 mM) by pipette, whereupon a noodle-like soft scaffold with an arbitrary length formed. After removal of the metal chloride solution, the soft scaffold was washed three times with Milli-Q water, and the resulting soft scaffold was used directly for POM experiments. A soft scaffold for SEM analysis was directly prepared on conductive carbon adhesive tape and air-dried for 48 h before measurement.

### SLS

The scattering intensity of the samples was determined by SLS measurements using a Wyatt Technology DynaPro NanoStar. The scattering intensity was recorded as a parameter signifying the assembly size since the objects in solution were anisotropic, and the models used by Wyatt software are fitted to spherical objects. To determine the CAC of **DA_6_**, **DA_7_**, and **DA_11_**, the scattering intensity of solutions of **DA_6_** (1.0 × 10^−4^–0.5 mM), **DA_7_** (1.0 × 10^−4^–0.5 mM), and **DA_11_** (1.0 × 10^–4^–0.1 mM) was recorded at 20 °C. This scattering rate was normalized by the concentration of the solution to yield the molar scattering intensity. Ten replications were performed, and the data was averaged to show the molar scattering intensity and the error standard deviation.

### WAXD

WAXD of DA scaffolds was measured on a sapphire substrate (φ = 2.0 cm) using a Rigaku NANOPIX equipped with a HyPix-6000 (Rigaku) detector. The scattering vector (*q* = 4⋅π⋅sin(θ)/λ), scattering angle θ, and the position of the incident X-ray beam on the detectors were calibrated using several orders of layer reflections from silver behenate (*d* = 58.380 Å), where λ refers to the wavelength of the X-ray beam (Cu Kα irradiation, 1.54 Å). The sample-to-detector distance was ≈100 mm. The obtained diffraction patterns were integrated along the Debye–Scherrer ring to afford 1D intensity data using the Rigaku 2DP software.

### Attachment of hBM-MSCs onto DA*_n_* scaffold surface

The preparation of **DA*_n_*** stock solutions [61] and the fabrication of macroscopic scaffolds followed the standard methods outlined above. An aqueous solution of **DA*_n_*** (5.0 wt %) was manually added to a calcium chloride solution (150 mM) in a bioinert surface dish to fabricate the macroscopic scaffold. After removal of the calcium chloride solution, the obtained **DA*_n_*** macroscopic scaffold was washed three times with fresh Milli-Q water, followed by the addition of 0.5 mL of a growth medium consisting of minimum essential medium (MEM α, no phenol red, Gibco), 10% fetal bovine serum (FBS, Gibco), and 1% antibiotic–antimycotic (Gibco). Then, hBM-MSCs (Lonza) were added to the culture dish and cultured for 3 days at 37 °C and 5% CO_2_ in an incubator. After incubation, the **DA*_n_*** macroscopic scaffold with cells attached was used directly for fluorescent microscopy measurements.

## Supporting Information

File 1 Experimental details, supporting figures, and copies of spectra.

File 2 Monitoring of **DA_11_** under visible-light irradiation.

File 3 Monitoring of **DA_7_** under visible-light irradiation.

File 4 Monitoring of **DA_6_** under visible-light irradiation.

## Data Availability

The data that supports the findings of this study is available from the corresponding author upon reasonable request.
