# Supplementary material for: Supramolecular assemblies of amphiphilic donor–acceptor Stenhouse adducts as macroscopic soft scaffolds
Source: Beilstein J Org Chem. 2024 Jul 15;20:1590–603. doi: 10.3762/bjoc.20.142 (PMC11285068; doi:10.3762/bjoc.20.142)
Supplement: File 1 — Experimental details, supporting figures, and copies of spectra. [file Beilstein_J_Org_Chem-20-1590-s001.pdf]

BEILSTEIN JOURNAL OF ORGANIC CHEMISTRY

Supporting Information

for

Supramolecular assemblies of amphiphilic donor–acceptor Stenhouse adducts as macroscopic soft scaffolds

Ka-Lung Hung, Leong-Hung Cheung, Yikun Ren, Ming-Hin Chau, Yan-Yi Lam, Takashi Kajitani and Franco King-Chi Leung

Beilstein J. Org. Chem. **2024,** *20,* 1590–1603. doi:10.3762/bjoc.20.142

Experimental details, supporting figures, and copies of spectra

License and Terms: This is a supporting information file under the terms of the Creative Commons Attribution License (https://creativecommons.org/licenses/by/4.0). Please note that the reuse, redistribution and reproduction in particular requires that the author(s) and source are credited and that individual graphics may be subject to special legal provisions.
The license is subject to the *Beilstein Journal of Organic Chemistry* terms and conditions: (https://www.beilstein-journals.org/bjoc/terms)

Table of contents:

1. **Synthesis S2**
2. **Supporting figures S9**
3. **Copies of spectra S20**
4. **References S31**

1. Synthesis

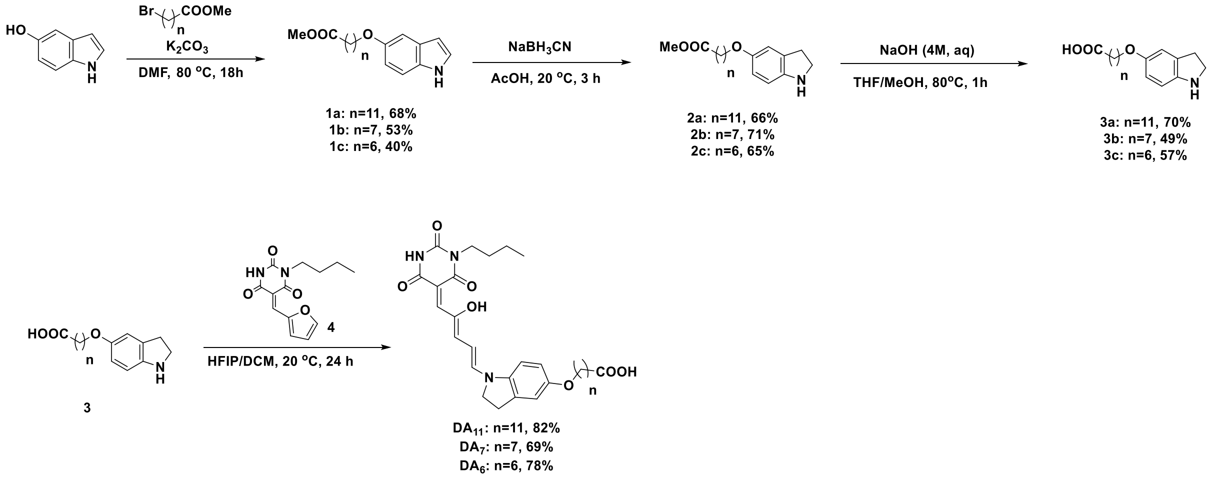

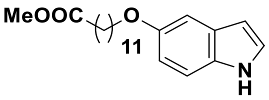

1a

Compound **1a**

A mixture of 5-hydroxyindole (509 mg, 3.8 mmol, 1.0 equiv), methyl 12-bromododecanoate (1.35 g, 4.56 mmol, 1.2 equiv) and potassium carbonate (1.06 g, 7.6 mmol, 2.0 equiv) in DMF (5 mL) was heated at 80 °C for 18 h. The reaction mixture was diluted with ethyl acetate (20 mL) after cooling to room temperature, washed with brine (1 × 20 mL), water (2 × 20 mL) and brine (1 × 20 mL) sequentially. The organic layer was dried over Na_2SO_4 and the filtrate was concentrated under reduced pressure. The crude was purified by flash column chromatography on SiO_2 (*n*-hexane/ethyl acetate = 5/1, R_f = 0.5) to afford compound **1a** (897 mg, 2.60 mmol, 68% yield) as a white solid.

^{1}H NMR (600 MHz, $CDCl_3$) δ 8.14 (s, 1H), 7.24 (d, J = 8.8 Hz, 1H), 7.14 (t, J = 2.9 Hz, 1H), 7.10 (d, J = 2.3 Hz, 1H), 6.85 (dd, J = 8.8, 2.4 Hz, 1H), 6.45 (t, J = 2.7 Hz, 1H), 3.99 (t, J = 6.6 Hz, 2H), 3.66 (s, 3H), 2.30 (t, J = 7.6 Hz, 2H), 1.79 (p, J = 6.8 Hz, 2H), 1.62 (q, J = 7.2 Hz, 2H), 1.50 – 1.42 (m, 2H), 1.37 – 1.27 (m, 12H).

^{13}C NMR (151 MHz, $CDCl_3$) δ 174.49, 153.63, 130.99, 128.32, 124.84, 112.94, 111.66, 103.48, 103.46, 102.29, 68.86, 51.51, 34.17, 29.60, 29.55, 29.52, 29.47, 29.28, 29.18, 26.17, 25.00.

HR-MS (ESI+) calculated for $C_{21}H_{32}NO_3$ $[M+H]^+$ m/z 346.2382, found 346.2383.

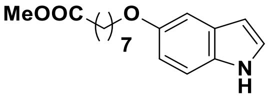

1b

Compound **1b**

A mixture of 5-hydroxyindole (1.08 g, 8.1 mmol, 1.0 equiv), methyl 7-bromoheptanoate (2.32 g, 9.7 mmol, 1.2 equiv) and potassium carbonate (2.25 g, 16.2 mmol, 2.0 equiv) in DMF (7 mL) was heated at 80 °C for 18 h. The reaction mixture was diluted with ethyl acetate (20 mL) after cooling to room temperature, washed with brine (1 × 20 mL), water (2 × 20 mL) and brine (1 × 20 mL) sequentially. The organic layer was dried over Na_2SO_4 and the filtrate was concentrated under reduced pressure. The crude was purified by flash column chromatography on SiO_2 (*n*-hexane/ethyl acetate = 5/1, R_f = 0.3) to afford compound **1b** (1.30 g, 4.5 mmol, 53% yield) as a white solid.

^{1}H NMR (600 MHz, CDCl3) δ 8.67 (s, 1H), 7.27 (d, J = 8.8 Hz, 1H), 7.23 (d, J = 2.7 Hz, 1H), 7.14 (d, J = 3.0 Hz, 1H), 6.99 (dd, J = 8.6, 2.6 Hz, 1H), 6.55 (d, J = 3.4 Hz, 1H), 4.06 (t, J = 6.5 Hz, 2H), 3.75 (d, J = 2.6 Hz, 3H), 2.40 (t, J = 7.6 Hz, 2H), 1.87 (q, J = 7.2 Hz, 2H), 1.72 (p, J = 7.3 Hz, 2H), 1.54 (q, J = 7.5 Hz, 2H), 1.47 – 1.38 (m, 5H).

^{13}C NMR (151 MHz, CDCl3) δ 174.64, 153.53, 131.30, 128.46, 125.26, 112.73, 111.99, 103.48, 101.97, 68.81, 51.56, 34.13, 29.56, 29.18, 26.10, 25.01.

HR-MS (ESI+) calculated for $C_{18}H_{26}NO_3$ $[M+H]^+$ m/z 290.1756, found 290.1755.

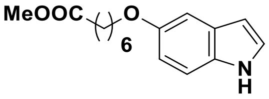

1c

Compound **1c**

A mixture of 5-hydroxyindole (554 mg, 4.2 mmol, 1.0 equiv), methyl 7-bromoheptanoate (1.11 g, 5.0 mmol, 1.2 equiv) and potassium carbonate (1.22 g, 8.8 mmol, 2.0 equiv) in DMF (5 mL) was heated at 80 °C for 18 h. The reaction mixture was diluted with ethyl acetate (20 mL) after cooling to room temperature, washed with brine (1 × 20 mL), water (2 × 20 mL) and brine (1 × 20 mL) sequentially. The organic layer was dried over Na_2SO_4 and the filtrate was concentrated under reduced pressure. The crude was purified by flash column chromatography on SiO_2 (*n*-hexane/ethyl acetate = 5/1, R_f = 0.5) to afford compound **1c** (458 mg, 1.66 mmol, 40% yield) as a white solid.

^{1}H NMR (600 MHz, $CDCl_3$) δ 8.07 (s, 1H), 7.27 (d, *J* = 8.7 Hz, 1H), 7.17 (t, *J* = 2.8 Hz, 1H), 7.10 (d, *J* = 2.4 Hz, 1H), 6.85 (dd, *J* = 8.8, 2.4 Hz, 1H), 6.47 (ddd, *J* = 3.1, 2.0, 0.9 Hz, 1H), 3.99 (t, *J* = 6.5 Hz, 2H), 3.67 (s, 3H), 2.33 (t, *J* = 7.5 Hz, 2H), 1.84 – 1.77 (m, 2H), 1.67 (p, *J* = 7.6 Hz, 2H), 1.51 (dtd, *J* = 9.4, 7.4, 5.5 Hz, 2H), 1.44 – 1.36 (m, 2H).

^{13}C NMR (151 MHz, $CDCl_3$) δ 174.29, 153.58, 130.94, 128.28, 124.77, 112.92, 111.61, 103.45, 102.35, 68.61, 51.50, 34.04, 29.30, 28.94, 25.83, 24.90.

HR-MS (ESI+) calculated for C16H22NO3 $[M+H]^+$ m/z 276.1600, found 276.1595.

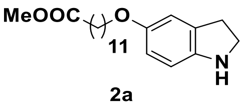

2a

Compound **2a**

To a solution of compound **1a** (217 mg, 0.63 mmol, 1.0 equiv) in acetic acid (4 mL), sodium cyanoborohydride (119 mg, 1.89 mmol, 3.0 equiv) was added in one portion. The reaction mixture was stirred at 20 °C for 3 h. The reaction was quenched by adding H_2O (5 mL) in an ice bath. The pH of reaction mixture was adjusted to ≈ 7 by adding 4 M NaOH solution. The reaction mixture was extracted with ethyl acetate (3 × 15 mL) and the combined organic layer was washed with brine (1 × 15 mL). The organic layer was dried over Na_2SO_4 and the filtrate was concentrated under reduced pressure. The residue was purified by flash column chromatography on SiO_2 (*n*-hexane/ethyl acetate = 2/1, R_f = 0.10) to afford compound **2a** (145 mg, 0.42 mmol, 66% yield) as an off-white solid.

^{1}H NMR (600 MHz, $CDCl_3$) δ 6.76 (s, 1H), 6.59 (d, *J* = 2.1 Hz, 2H), 3.87 (t, *J* = 6.6 Hz, 2H), 3.67 (s, 3H), 3.53 (t, *J* = 8.3 Hz, 2H), 3.00 (t, *J* = 8.3 Hz, 2H), 2.30 (t, *J* = 7.6 Hz, 2H), 1.73 (p, *J* = 6.8 Hz, 2H), 1.62 (p, *J* = 7.4 Hz, 2H), 1.42 (p, *J* = 7.4, 7.0 Hz, 2H), 1.35 – 1.26 (m, 12H).

^{13}C NMR (151 MHz, $CDCl_3$) δ 174.39, 153.13, 145.09, 131.13, 113.13, 112.39, 110.23, 68.99, 51.46, 47.80, 34.14, 30.49, 29.56, 29.52, 29.48, 29.44, 29.42, 29.26, 29.16, 26.09, 24.97.

HR-MS (ESI+) calculated for $C_{21}H_{34}NO_3$ $[M+H]^+$ m/z 348.2539, found 348.2539.

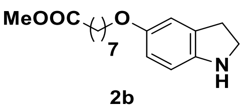

2b

Compound **2b**

To a solution of compound **1b** (1.7 g, 5.9 mmol, 1.0 equiv) in acetic acid (6 mL), sodium cyanoborohydride (1.1 g, 17.6 mmol, 3.0 equiv) was added in one portion. The reaction mixture was stirred at 20 °C for 3 h. The reaction was quenched by adding H_2O (5 mL) in an ice bath. The pH of reaction mixture was adjusted to ≈ 7 by adding 4 M NaOH solution. The reaction mixture was extracted with ethyl acetate (3 × 15 mL) and the combined organic layer was washed with brine (1 × 15 mL). The organic layer was dried over Na_2SO_4 and the filtrate was concentrated under reduced pressure. The residue was purified by flash column chromatography on SiO_2 (*n*-hexane/ethyl acetate = 2/1, R_f = 0.15) to afford compound **2b** (1.2 g, 4.0 mmol, 71% yield) as a white solid.

^{1}H NMR (600 MHz, $CDCl_3$) δ 6.75 (s, 1H), 6.58 (d, *J* = 2.7 Hz, 2H), 3.87 (t, *J* = 6.2 Hz, 2H), 3.67 (d, *J* = 2.0 Hz, 3H), 3.51 (t, *J* = 8.0 Hz, 2H), 2.99 (t, *J* = 8.3 Hz, 2H), 2.31 (t, *J* = 7.3 Hz, 2H), 1.77 – 1.69 (m, 2H), 1.64 (p, *J* = 7.1 Hz, 2H), 1.45 (t, *J* = 7.7 Hz, 2H), 1.40 – 1.28 (m, 5H).

^{13}C NMR (151 MHz, $CDCl_3$) δ 174.57, 174.27, 153.18, 144.87, 131.25, 113.10, 112.30, 110.44, 68.82, 51.46, 47.70, 34.05, 30.46, 29.71, 29.39, 29.12, 29.07, 29.04, 28.99, 25.92, 25.84, 24.88.

HR-MS (ESI+) calculated for $C_{18}H_{28}NO_3$ $[M+H]^+$ m/z 292.1913, found 292.1912.

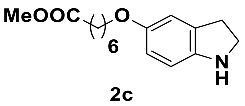

2c

Compound **2c**

To a solution of compound **1c** (164 mg, 0.59 mmol, 1.0 equiv) in acetic acid (6 mL), sodium cyanoborohydride (112 mg, 1.78 mmol, 3.0 equiv) was added in one portion. The reaction mixture was stirred at 20 °C for 3 h. The reaction was quenched by adding H_2O (5 mL) in an ice bath. The pH of reaction mixture was adjusted to ≈ 7 by adding 4 M NaOH solution. The reaction mixture was extracted with ethyl acetate (3 × 15 mL) and the combined organic layer was washed with brine (1 × 15 mL). The organic layer was dried over Na_2SO_4 and the filtrate was concentrated under reduced pressure. The residue was purified by flash column chromatography on SiO_2 (*n*-hexane/ethyl acetate = 2/1, R_f = 0.15) to afford compound **2c** (107 mg, 0.38 mmol, 65% yield) as a white solid.

^{1}H NMR (600 MHz, $CDCl_3$) δ 6.73 (d, *J* = 1.8 Hz, 1H), 6.59 – 6.51 (m, 2H), 3.85 (t, *J* = 6.5 Hz, 2H), 3.65 (s, 3H), 3.49 (q, *J* = 9.8, 8.3 Hz, 3H), 2.97 (t, *J* = 8.3 Hz, 2H), 2.31 (t, *J* = 7.5 Hz, 2H), 1.72 (dt, *J* = 14.5, 6.6 Hz, 2H), 1.64 (p, *J* = 7.5 Hz, 2H), 1.50 – 1.41 (m, 2H), 1.41 – 1.33 (m, 2H).

^{13}C NMR (151 MHz, $CDCl_3$) δ 174.14, 152.84, 145.36, 130.99, 113.01, 112.29, 110.01, 68.68, 51.42, 47.75, 33.95, 30.43, 29.26, 28.88, 25.75, 24.85.

HR-MS (ESI+) calculated for $C_{16}H_{24}NO_3$ $[M+H]^+$ m/z 278.1756, found 278.1755.

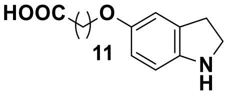

3a

Compound **3a**

To a solution of compound **2a** (443 mg, 1.27 mmol) in methanol (2 mL) and tetrahydrofuran (2 mL), an aqueous NaOH solution (4 M, 1 mL) was added and the reaction mixture was then heated at 80 °C for 1 h. After cooling to room temperature, volatile solvents were removed under reduced pressure. The pH of reaction mixture was adjusted to ≈ 7 by adding 1 M HCl solution that off-white precipitate formed. The filtered precipitate was washed with H_2O (10 mL) to afford compound **3a** as an off-white solid (297 mg, 0.89 mmol, 70% yield).

^{1}H NMR (400 MHz, 9:1 $CDCl_3$ with 0.03% v/v TMS:CD_3OD) δ 6.77 (s, 1H), 6.69 – 6.58 (m, 2H), 3.89 (t, *J* = 6.6 Hz, 2H), 3.51 (t, *J* = 8.2 Hz, 2H), 3.02 (t, *J* = 8.3 Hz, 2H), 2.29 (t, *J* = 7.6 Hz, 2H), 1.74 (q, *J* = 7.0 Hz, 2H), 1.61 (t, *J* = 7.3 Hz, 2H), 1.43 (t, *J* = 7.5 Hz, 2H), 1.36 – 1.26 (m, 12H).

^{13}C NMR (151 MHz, 9:1 $CDCl_3$ with 0.03% v/v TMS:CD_3OD) δ 177.00, 153.68, 144.14, 131.74, 113.30, 112.26, 111.33, 69.04, 47.47, 34.22, 30.45, 29.50, 29.47, 29.41, 29.37, 29.34, 29.26, 29.14, 25.99, 24.95.

HR-MS (ESI+) calculated for $C_{20}H_{32}NO_3$ $[M+H]^+$ m/z 334.2382, found 334.2384.

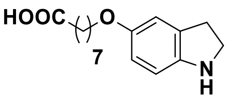

3b

Compound **3b**

To a solution of compound **2b** (107 mg, 0.35 mmol, 1.0 equiv) in methanol (2 mL) and tetrahydrofuran (2 mL), an aqueous NaOH solution (4 M, 1 mL) was added and the reaction mixture was then heated at 80 °C for 1 h. After cooling to room temperature, volatile solvents were removed under reduced pressure. The pH of reaction mixture was adjusted to ≈ 7 by adding 1 M HCl solution that off-white precipitate formed. The filtered precipitate was washed with H_2O (10 mL) to afford compound **3b** as an off-white solid (49.5 mg, 0.17 mmol, 49% yield).

^{1}H NMR (600 MHz, DMSO) δ 6.68 (d, *J* = 2.5 Hz, 1H), 6.48 (dd, *J* = 8.3, 2.5 Hz, 1H), 6.40 (d, *J* = 8.3 Hz, 1H), 3.80 (t, *J* = 6.5 Hz, 2H), 2.84 (t, *J* = 8.4 Hz, 2H), 2.19 (t, *J* = 7.4 Hz, 2H), 1.63 (p, *J* = 6.7 Hz, 2H), 1.49 (p, *J* = 7.3 Hz, 2H), 1.36 (td, *J* = 8.8, 8.1, 4.3 Hz, 2H), 1.33 – 1.25 (m, 4H).

^{13}C NMR (151 MHz, DMSO) δ 175.00, 151.81, 146.84, 130.77, 113.22, 112.48, 109.34, 68.53, 47.39, 34.14, 30.34, 29.34, 28.99, 28.98, 25.92, 24.93.

HR-MS (ESI+) calculated for $C_{17}H_{26}NO_3$ $[M+H]^+$ m/z 278.1756, found 278.1755.

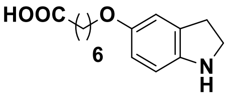

3c

Compound **3c**

To a solution of compound **2c** (100 mg, 0.36 mmol, 1.0 equiv.) in methanol (2 mL) and tetrahydrofuran (2 mL), an aqueous NaOH solution (4 M, 1 mL) was added and the reaction mixture was then heated at 80 °C for 1 h. After cooling to room temperature, volatile solvents were removed under reduced pressure. The pH of reaction mixture was adjusted to ≈ 7 by adding 1 M HCl solution that off-white precipitate formed. The filtered precipitate was washed with H_2O (10 mL) to afford compound **3c** as an off-white solid (54.1 mg, 0.20 mmol, 57% yield).

^{1}H NMR (600 MHz, DMSO) δ 6.68 (d, *J* = 2.2 Hz, 1H), 6.48 (dd, *J* = 8.3, 2.4 Hz, 1H), 6.40 (d, *J* = 8.3 Hz, 1H), 3.80 (t, *J* = 6.5 Hz, 2H), 2.84 (t, *J* = 8.4 Hz, 2H), 2.20 (t, *J* = 7.4 Hz, 2H), 1.63 (p, *J* = 6.6 Hz, 2H), 1.50 (p, *J* = 7.4 Hz, 2H), 1.37 (dt, *J* = 14.8, 7.1 Hz, 2H), 1.31 (q, *J* = 8.5, 8.0 Hz, 2H).

^{13}C NMR (151 MHz, DMSO) δ 174.54, 151.36, 146.40, 130.33, 112.77, 112.03, 108.89, 68.03, 46.95, 33.63, 29.90, 28.82, 28.36, 25.35, 24.49.

HR-MS (ESI+) calculated for $C_{15}H_{22}NO_3$ $[M+H]^+$ m/z 264.1600, found 264.1593.

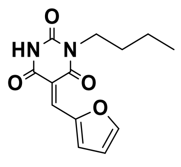

4

Compound **4** was synthesized as previously reported[1, 2].

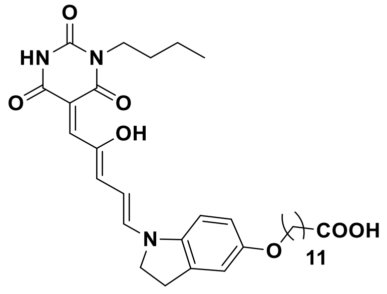

DA_{11}

DA$_{11}$

Compound **3 (**26.34 mg, 0.079 mmol, 1.0 equiv**)** and compound **4** (20.71 mg, 0.079 mmol, 1.0 equiv) were suspended in dichloromethane (0.8 mL). After adding hexafluoro-2-propanol (0.2 mL), the reaction mixture was stirred at 20 °C for 24 h under air. Undissolved solid was filtered off and washed with THF solution (3 mL). The filtrate was concentrated under reduced pressure. The residue was dissolved in THF (1 mL). After introducing *n*-hexane (10 mL), the recrystallized **DA$_{11}$** (38.7 mg, 0.065 mmol, 82% yield) was obtained as a black solid.

^{1}H NMR (600 MHz, DMSO) δ 12.72 (s, 1H), 12.00 (s, 1H), 10.93 (s, 1H), 8.55 (d, *J* = 11.7 Hz, 1H), 7.46 (dd, *J* = 9.0, 6.5 Hz, 1H), 7.09 (d, *J* = 12.7 Hz, 1H), 6.99 (d, *J* = 2.5 Hz, 2H), 6.94 (dd, *J* = 8.9, 2.5 Hz, 2H), 6.82 (d, *J* = 3.6 Hz, 1H), 6.14 (t, *J* = 11.9 Hz, 2H), 4.25 (t, *J* = 7.6 Hz, 3H), 3.97 (t, *J* = 6.5 Hz, 3H), 3.81 (s, 1H), 3.73 (dt, *J* = 15.0, 7.6 Hz, 3H), 3.25 (t, *J* = 7.7 Hz, 4H), 2.19 (td, *J* = 7.4, 3.8 Hz, 4H), 1.70 (p, *J* = 6.7 Hz, 3H), 1.63 (s, 1H), 1.53 – 1.42 (m, 8H), 1.39 (q, *J* = 7.4 Hz, 4H), 1.28 (dtt, *J* = 29.3, 14.4, 7.7 Hz, 14H), 0.92 – 0.85 (m, 6H).

HR-MS (ESI–) calculated for $C_{33}H_{44}N_3O_7$ [M–H]$^-$ m/z 594.3179, found 594.3178.

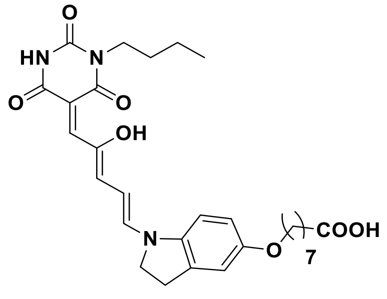

DA_7

DA$_7$

Compound **3b** (25.0 mg, 0.09 mmol, 1.0 equiv) and compound **4** (22.5 mg, 0.09 mmol, 1.0 equiv) were suspended in dichloromethane (0.8 mL). After adding hexafluoro-2-propanol (0.2 mL), the reaction mixture was stirred at 20 °C for 24 h under air. Undissolved solid was filtered off and washed with THF solution (3 mL). The filtrate was concentrated under reduced pressure. The residue was dissolved in THF (1 mL). After introducing *n*-hexane (10 mL), the recrystallized **DA$_7$** (65.6 mg, 0.12 mmol, 69% yield) was obtained as a green solid.

^{1}H NMR (600 MHz, $(CD_3)_2SO$) δ (ppm) 8.51 (dd, J = 23.2, 11.4 Hz, 1H), 7.46 (d, J = 8.8 Hz, 1H), 7.09 (d, J = 12.6 Hz, 1H), 7.01 – 6.90 (m, 2H), 6.82 (s, 1H), 6.14 (t, J = 11.9 Hz, 1H), 4.24 (t, J = 7.3 Hz, 2H), 3.97 (t, J = 6.4 Hz, 2H), 3.72 (dd, J = 14.6, 7.3 Hz, 2H), 2.19 (t, J = 7.3 Hz, 4H), 1.74 – 1.66 (m, 3H), 1.52 – 1.43 (m, 6H), 1.32 (dt, J = 20.0, 11.7 Hz, 17H).

HR-MS (ESI–) calculated for $C_{29}H_{36}N_3O_7$ $[M–H]^-$ m/z 538.2553, found 538.2552.

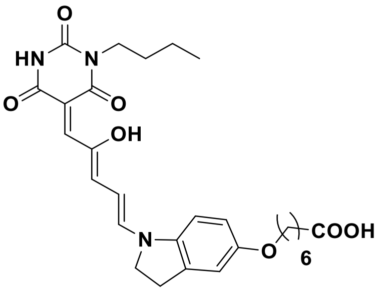

DA$_6$

DA$_6$

Compound **3c** (35 mg, 0.13 mmol, 1.0 equiv.) and compound **4** (34.9 mg, 0.13 mmol, 1.0 equiv) were suspended in dichloromethane (0.8 mL). After adding hexafluoro-2-propanol (0.2 mL), the reaction mixture was stirred at 20 °C for 24 h under air. Undissolved solid was filtered off and washed with THF solution (3 mL). The filtrate was concentrated under reduced pressure. The residue was dissolved in THF (1 mL). After introducing *n*-hexane (10 mL), the recrystallized **DA$_6$** (54.7 mg, 0.10 mmol, 78% yield) was obtained as a green solid.

^{1}H NMR (400 MHz, $(CD_3)_2SO$) δ (ppm) 8.51 (t, J = 13.0 Hz, 1H), 7.46 (d, J = 8.9 Hz, 1H), 7.09 (d, J = 12.7 Hz, 1H), 7.01 – 6.89 (m, 2H), 6.82 (s, 1H), 6.14 (t, J = 12.2 Hz, 1H), 4.24 (t, J = 7.1 Hz, 2H), 3.97 (t, J = 6.4 Hz, 3H), 2.50 (d, J = 1.4 Hz, 6H), 2.21 (t, J = 7.3 Hz, 3H), 1.80 – 1.60 (m, 3H), 1.58 – 1.19 (m, 15H).

HR-MS (ESI–) calculated for $C_{28}H_{34}N_3O_7$ $[M–H]^-$ m/z 524.2397, found 524.2392.

2. Supporting figures

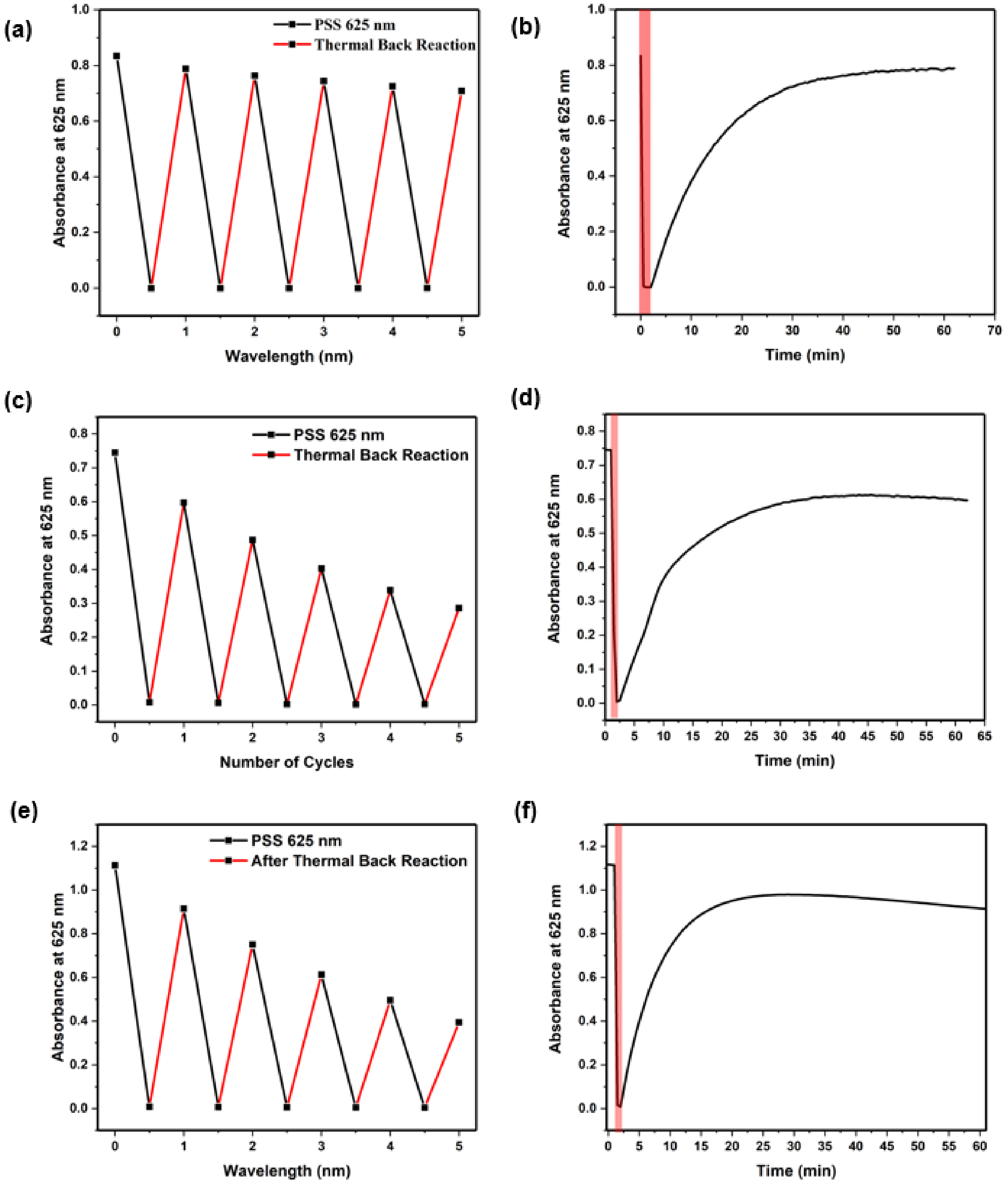

Figure S1. Multiple photoswitching cycles of (a) **DA_{11}**, (c) **DA_{7}**, (e) **DA_{6}**, alternative irradiation at 625 nm (black) over 1 min and thermal back reaction at 20 °C over 60 min (blue). Time-course of the photoisomerization process of (b) **DA_{11}**, (d) **DA_{7}**, (f) **DA_{6}**, in MilliQ water, monitored at λ_{max} 625 nm (red area: white-light irradiation period). Irradiation of UV–vis sample was carried out at 20 °C using a Thorlabs model M625F2 high-power LED (625 nm, 1.0 A) positioned at a distance of 1 cm from the sample.

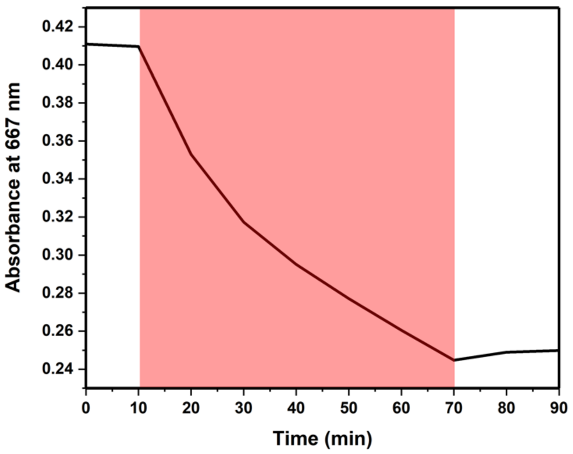

Figure S2. Time-course of the photoisomerization process of $\mathbf{DA_{11}}$ in MilliQ water, monitored at λ_{max} 667 nm (red area: white-light irradiation period). Irradiation of UV–vis samples was carried out at 20 °C using a light guide equipped BBZM-I xenon light source (380–800 nm, 300 W) positioned at a distance of 1 cm from the sample.

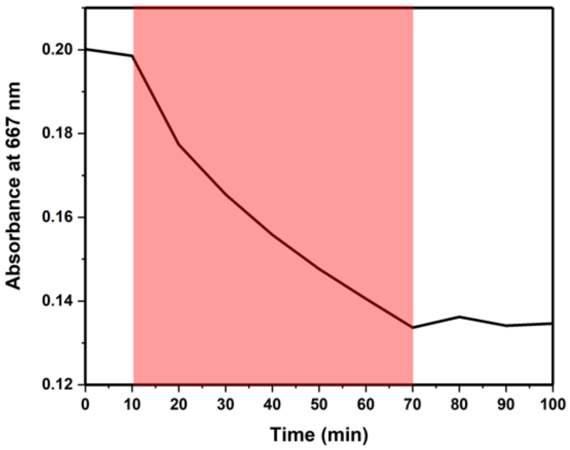

Figure S3. Time-course of the photoisomerization process of $\mathbf{DA_{7}}$ in MilliQ water, monitored at λ_{max} 667 nm (red area: white-light irradiation period). Irradiation of UV–vis samples was carried out at 20 °C using a light guide equipped BBZM-I xenon light source (380–800 nm, 300 W) positioned at a distance of 1 cm from the sample.

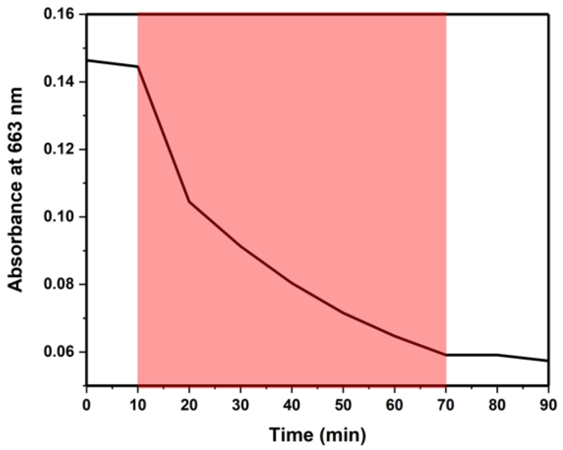

Figure S4. Time-course of the photoisomerization process of $\mathbf{DA_6}$ in MilliQ water, monitored at λ_{max} 663 nm (red area: white-light irradiation period). Irradiation of UV–vis samples was carried out at 20 °C using a light guide equipped BBZM-I xenon light source (380–800 nm, 300 W) positioned at a distance of 1 cm from the sample.

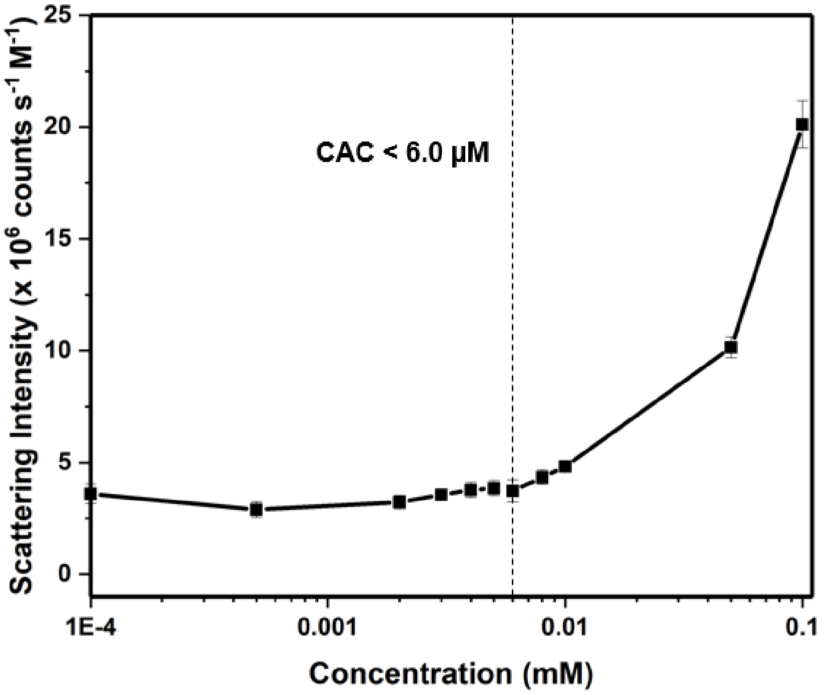

Figure S5. Static light scattering assay for determination of critical aggregation concentration of **DA**$_{11}$ (concentration: 1.0×10^{-4} to 0.1 mM).

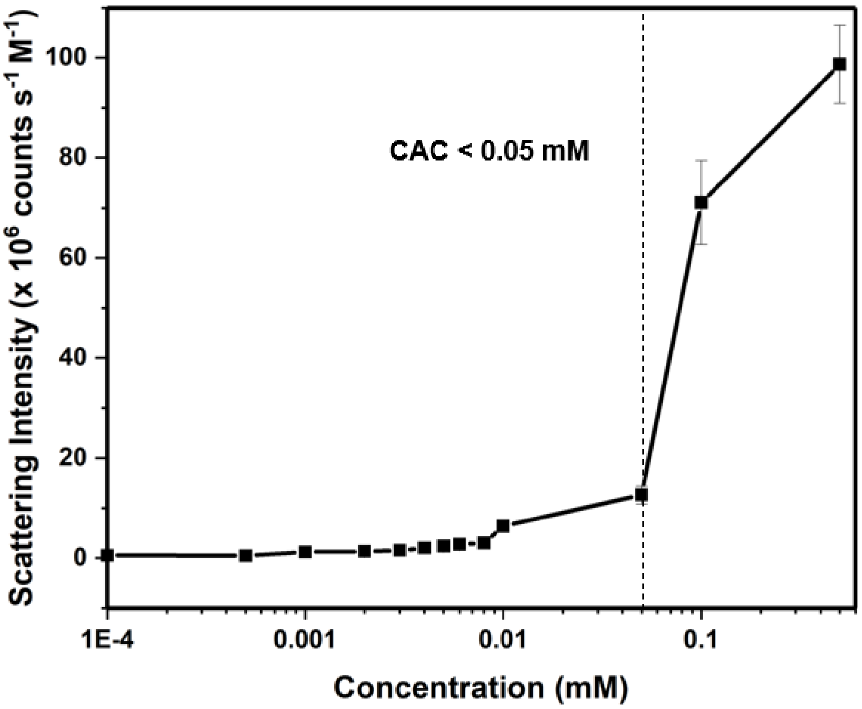

Figure S6. Static light scattering assay for determination of critical aggregation concentration of **DA**$_7$ (concentration: 1.0 × 10^{-4} to 0.5 mM).

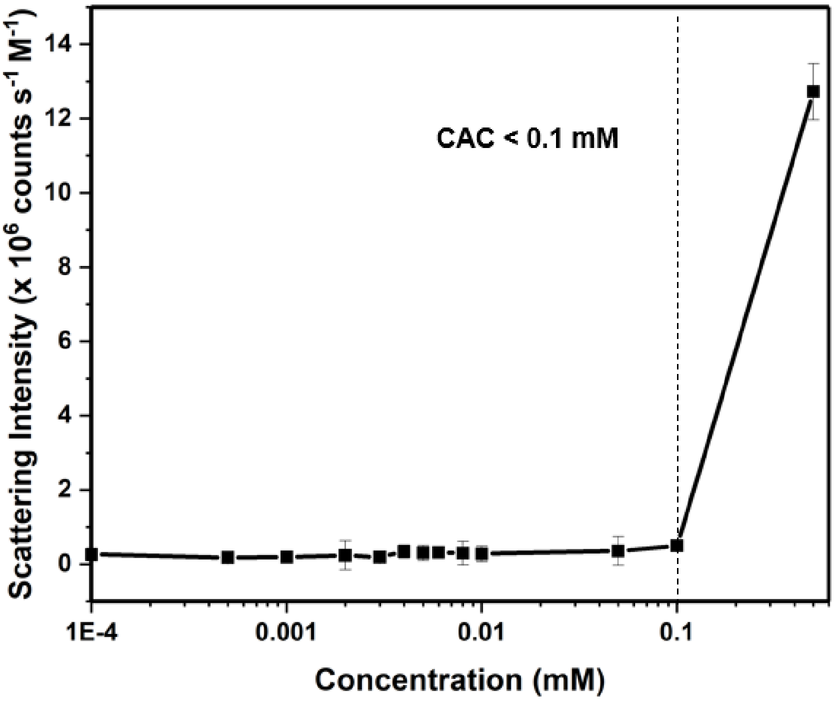

Figure S7. Static light scattering assay for determination of critical aggregation concentration of **DA$_6$** (concentration: 1.0×10^{-4} to 0.5 mM).

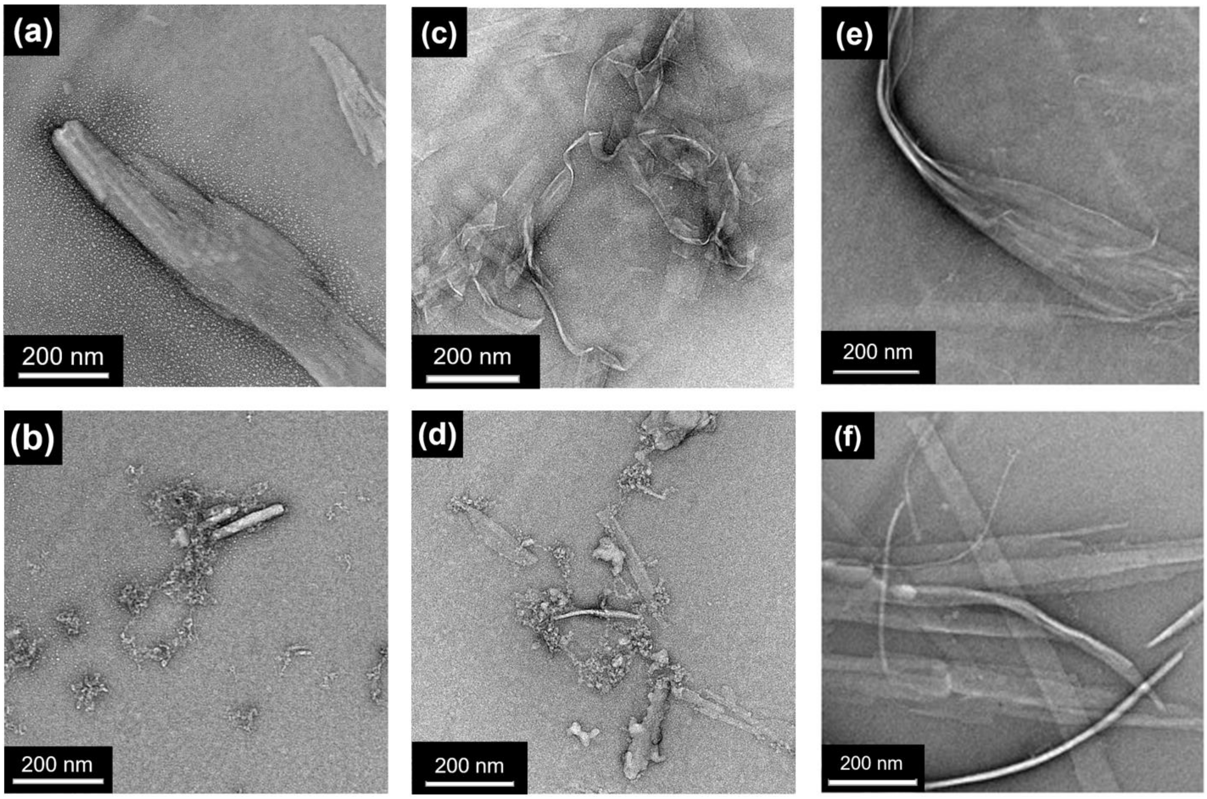

Figure S8. Representative TEM images of freshly prepared aqueous solutions before irradiation of (a) **DA$_{11}$** at 0.25 wt % (4.1 mM), (c) **DA$_7$** at 0.25 wt % (4.5 mM) and (e) **DA$_6$** at 0.25 wt % (4.6 mM). The solutions of (b) **DA$_{11}$**, (d) **DA$_7$**, (f) **DA$_6$** was irradiated with white-light irradiation over 60 min at 20 °C.

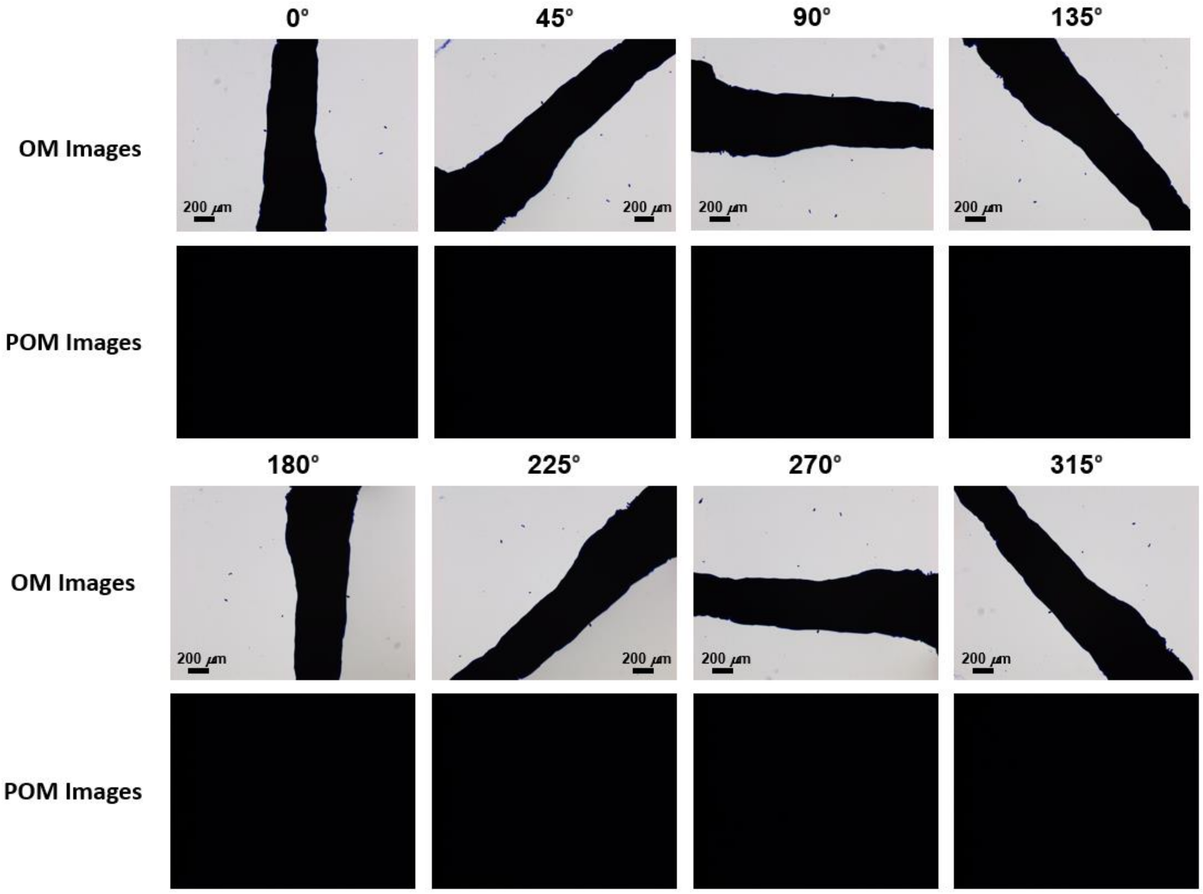

Figure S9. Optical microscopic images of a macroscopic soft scaffold composed of $\mathbf{DA_{11}}$ prepared from a solution of $CaCl_2$ (150 mM) under crossed polarizers. The POM and OM images of the soft scaffold were tilted at 0°, 45°, 90°, 135°, 180°, 225°, 270° and 315° relative to the transmission axis of the analyzer. Scale bar 200 μm for all panels.

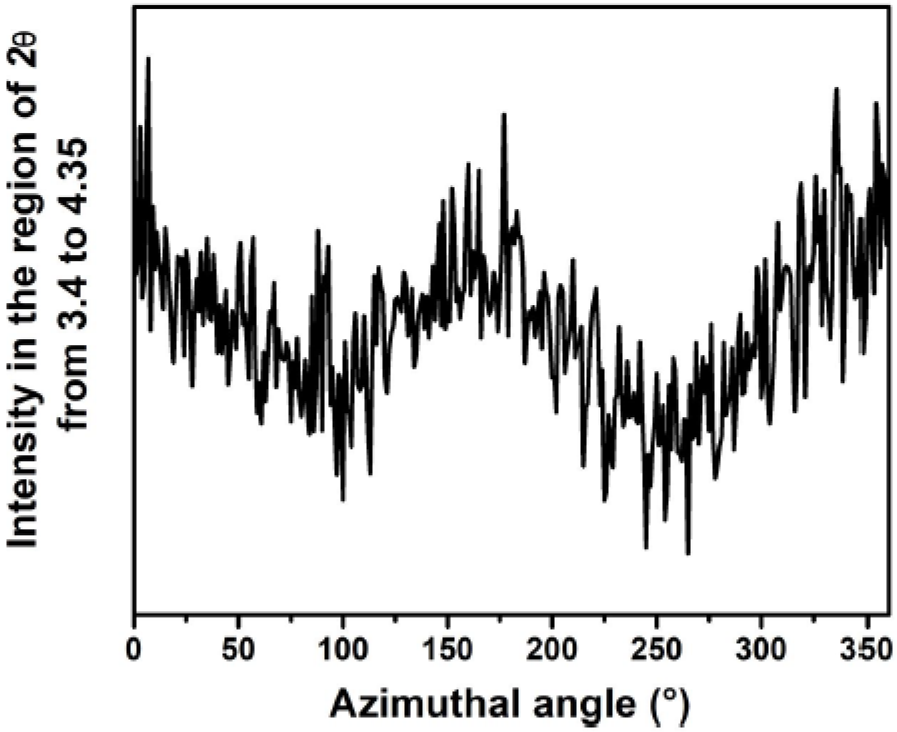

Figure S10. Full-width half maximum of *d* = 2.24 nm of the **DA$_{11}$** soft scaffold prepared from $CaCl_2$ solution (150 mM) at various azimuthal angle.

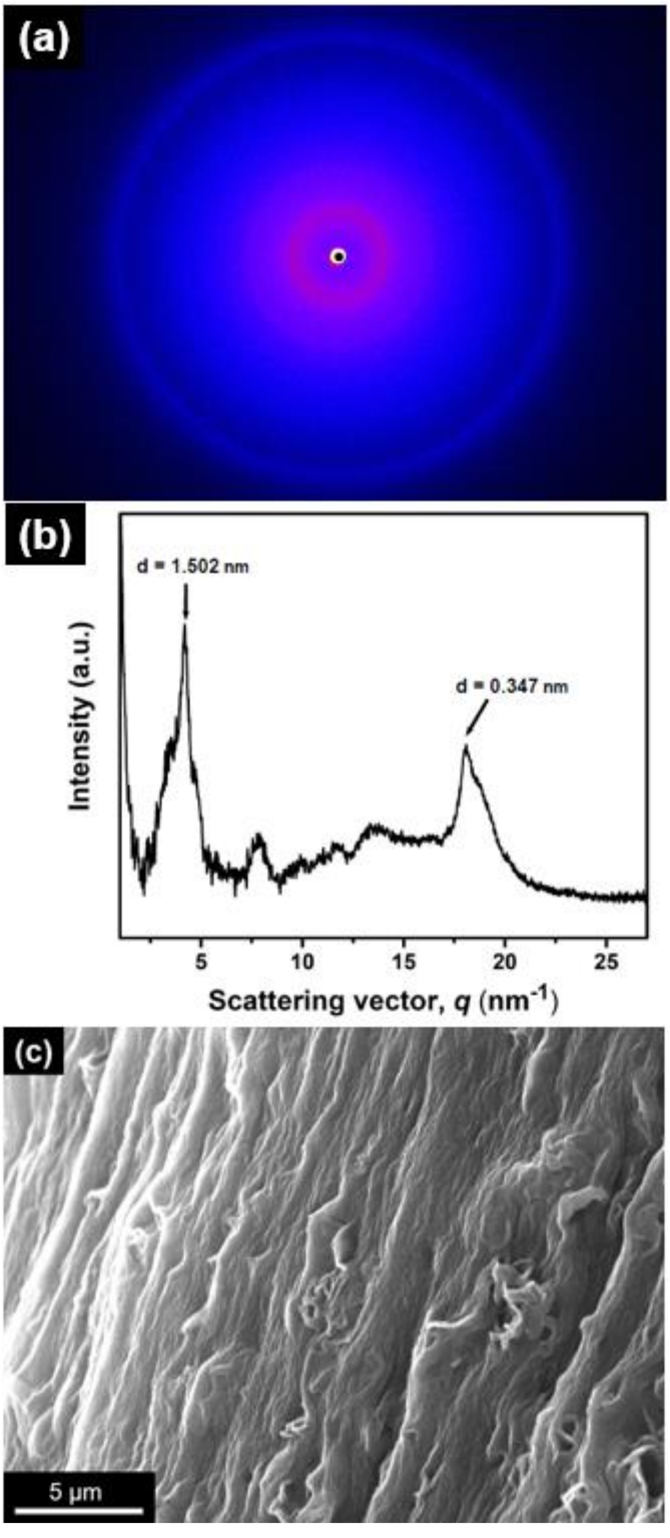

Figure S11. (a) 2D-WAXD image of **DA₇** macroscopic soft scaffold with 20 min X-ray exposure time. (b) 1D-WAXD pattern of **DA₇** macroscopic soft scaffold of 2D WAXD image in (a). The diffraction peaks at q = 7.5 nm^{-1} with d-spacing of 0.83 nm, which is originated from the high electron density moiety of the molecular structure of **DA₇** (EEE form) (calculated from Chem3D, ≈ 0.82 nm). (c) SEM image of a macroscopic scaffold composed of **DA₇** prepared from a solution of $CaCl_2$ (150 mM).

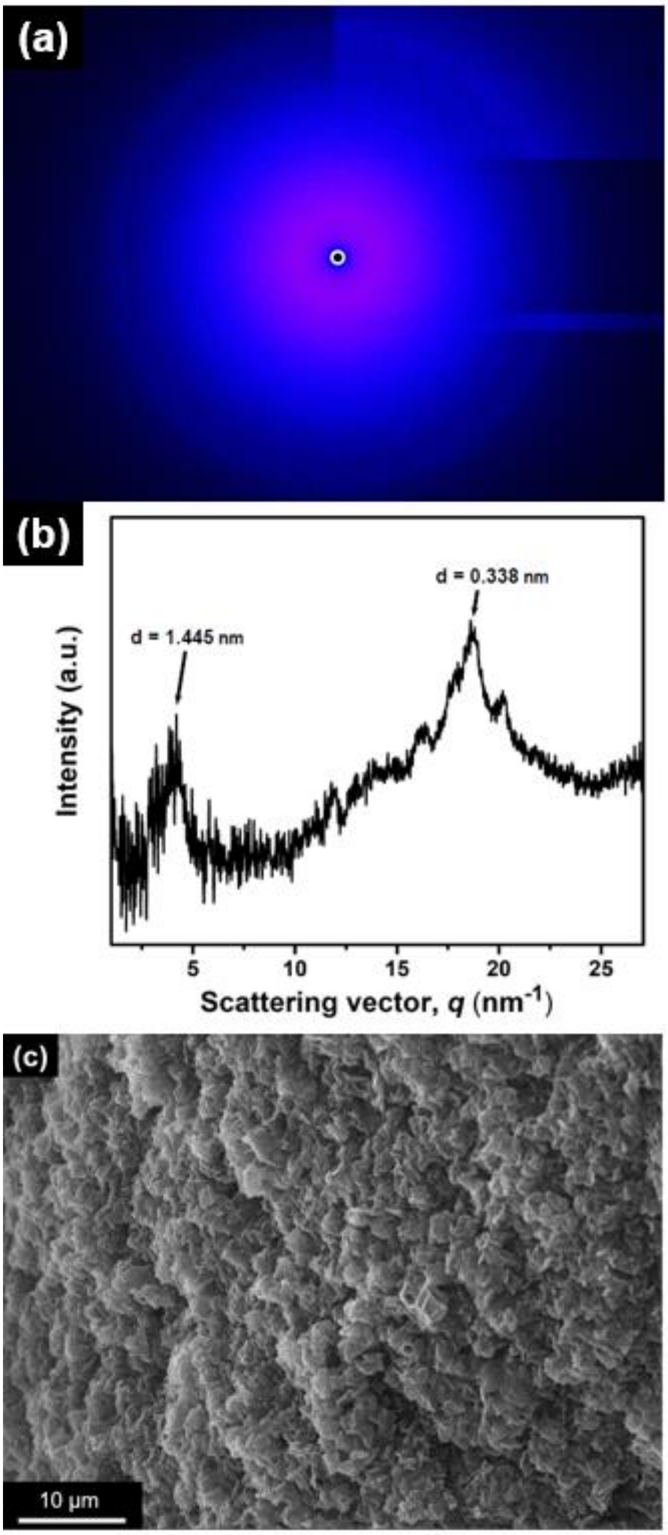

Figure S12. (a) 2D-WAXD image of **DA₆** macroscopic soft scaffold with 20 min X-ray exposure time. (b) 1D-WAXD pattern of **DA₆** macroscopic soft scaffold of 2D WAXD image in (a). (c) SEM image of a macroscopic scaffold composed of **DA₆** prepared from a solution of $CaCl_2$ (150 mM).

3. Copies of spectra

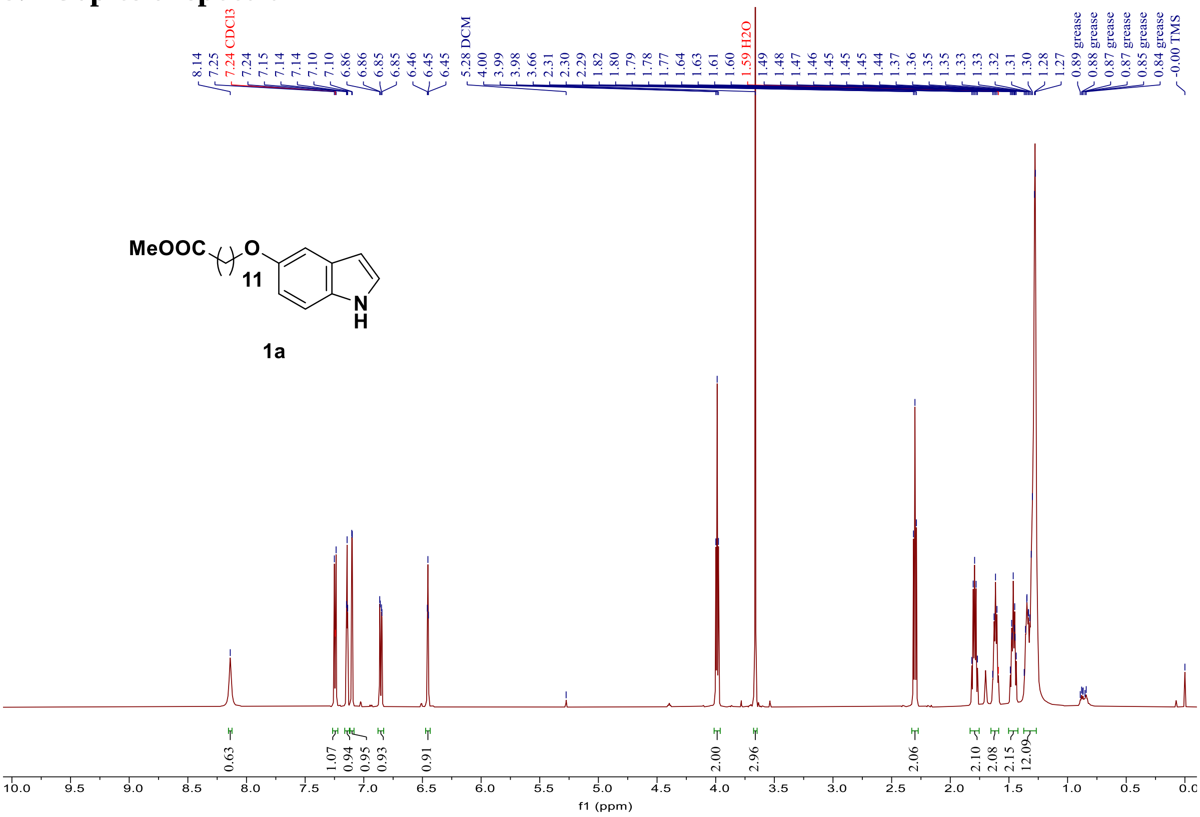

Figure S13. ^{1}H NMR spectrum (600 MHz, 25 °C) of compound **1a** in $CDCl_3$ with 0.03% v/v TMS.

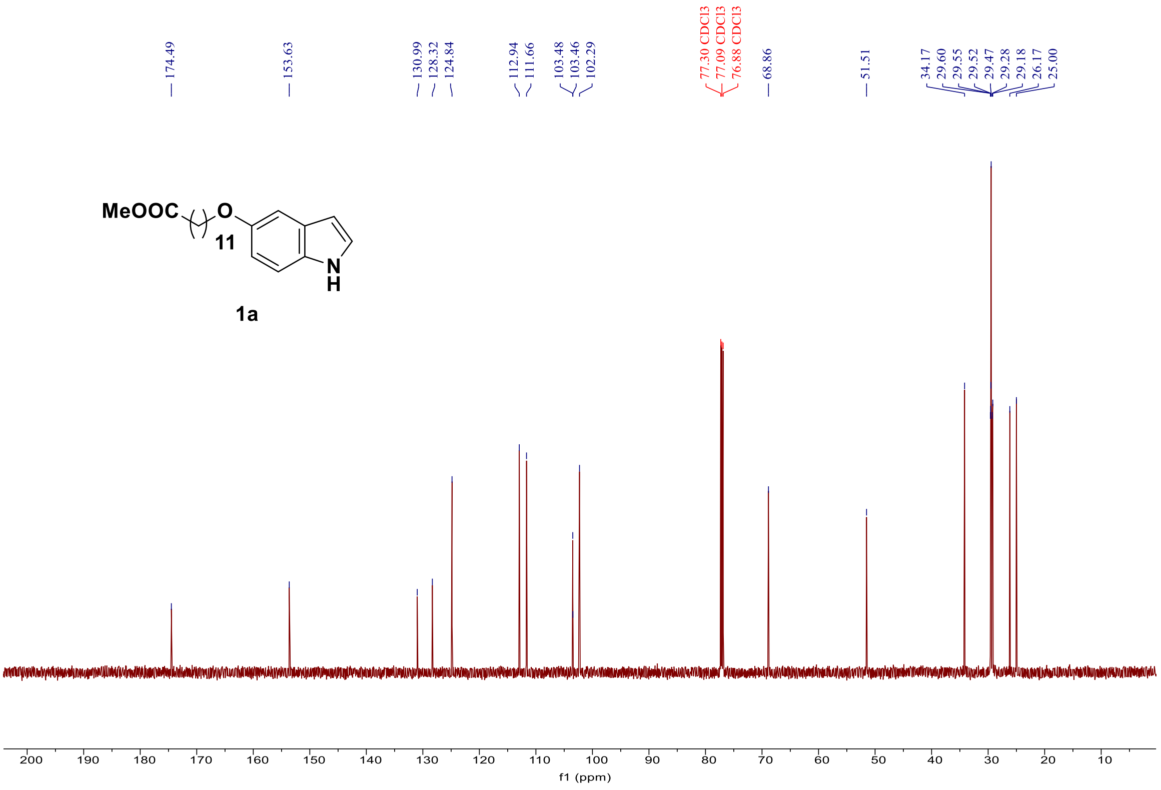

Figure S14. ^{13}C NMR spectrum (151 MHz, 25 °C) of compound **1a** in $CDCl_3$ with 0.03% v/v TMS.

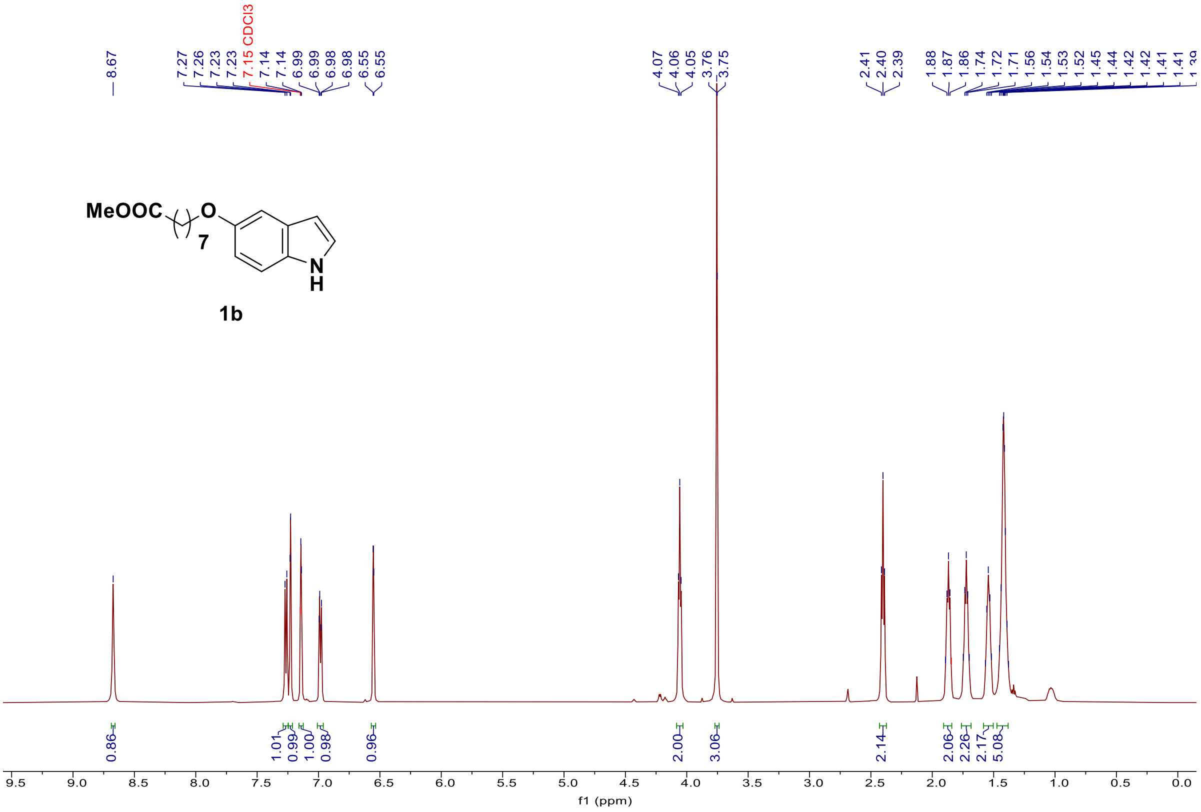

Figure S15. ^{1}H NMR spectrum (600 MHz, 25 °C) of compound **1b** in $CDCl_3$ with 0.03% v/v TMS.

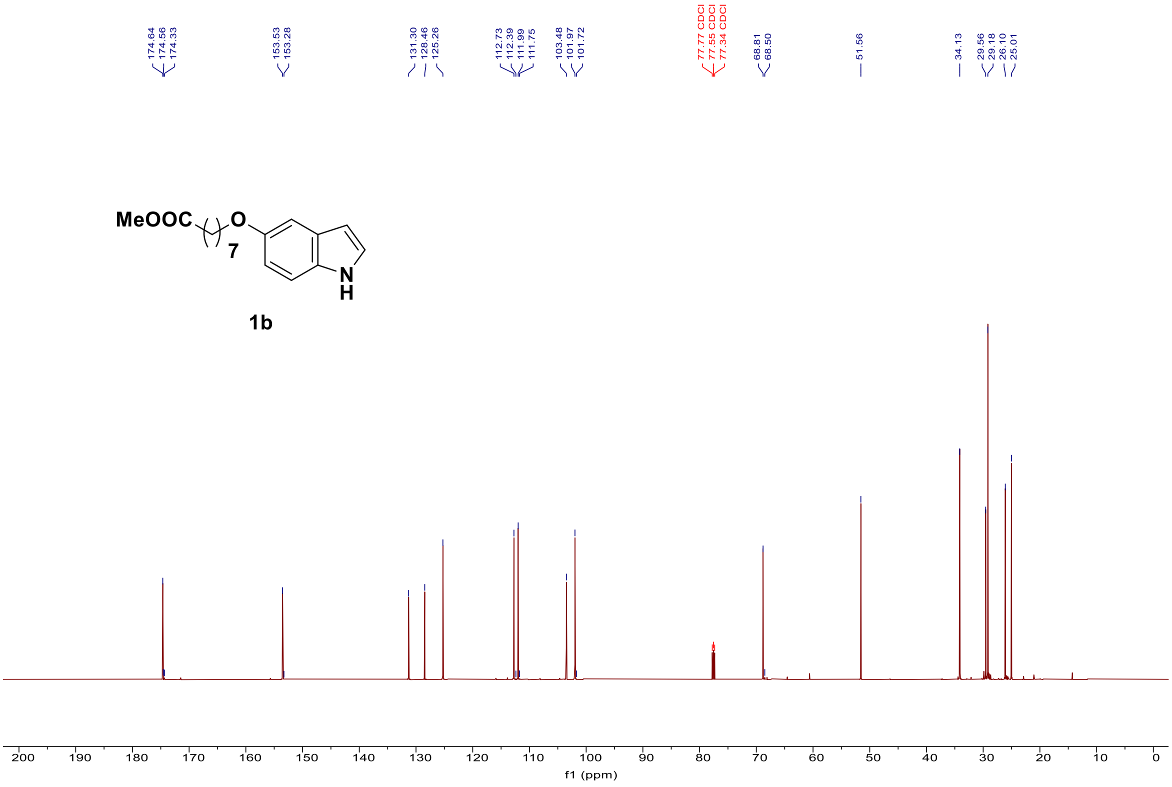

Figure S16. ^{13}C NMR spectrum (151 MHz, 25 °C) of compound **1b** in $CDCl_3$ with 0.03% v/v TMS.

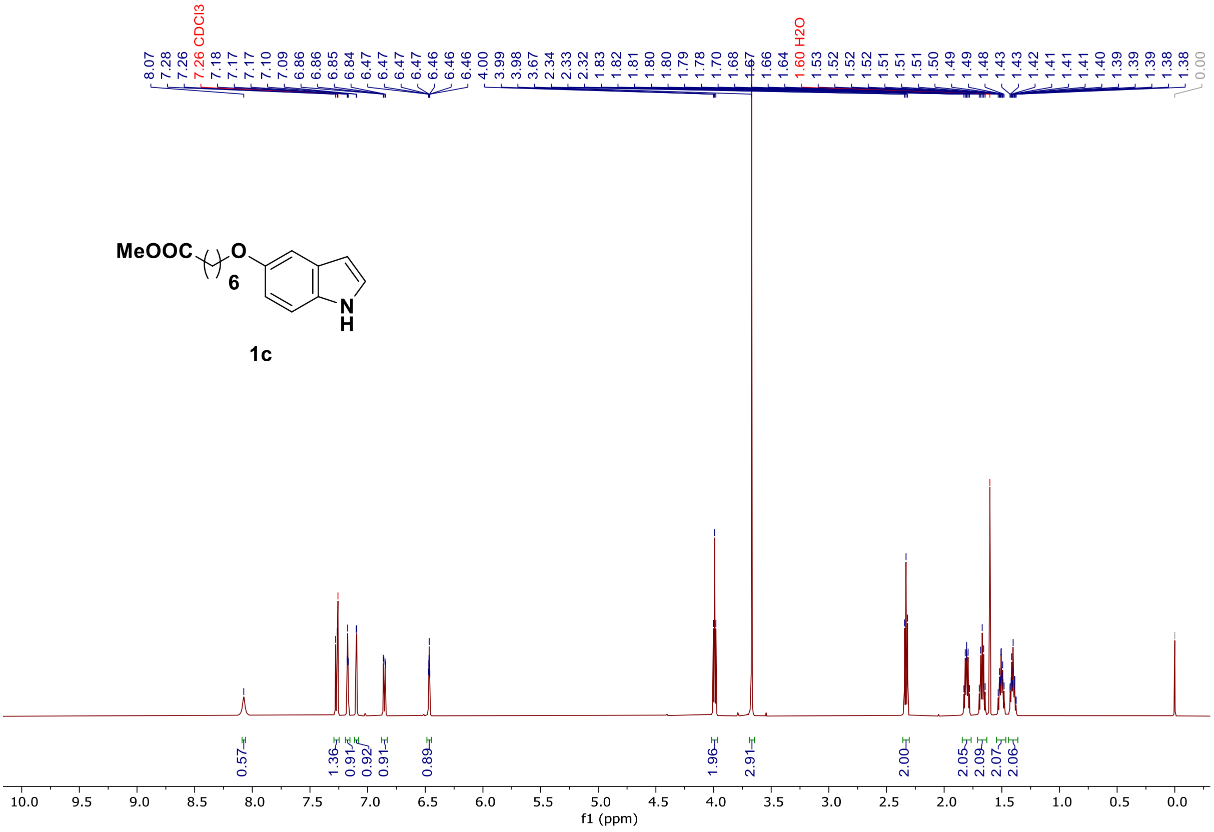

Figure S17. ^{1}H NMR spectrum (600 MHz, 25 °C) of compound **1c** in $CDCl_3$ with 0.03% v/v TMS.

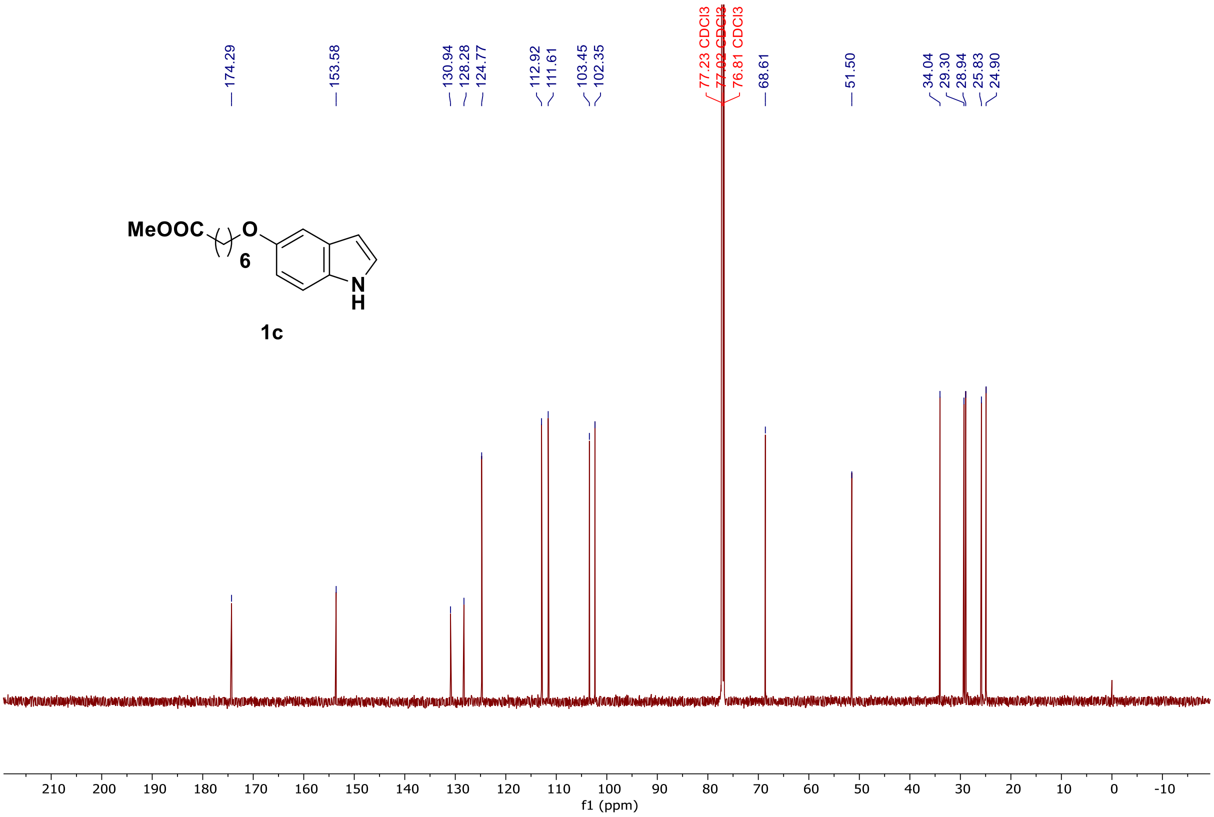

Figure S18. ^{13}C NMR spectrum (151 MHz, 25 °C) of compound **2a** in $CDCl_3$ with 0.03% v/v TMS.

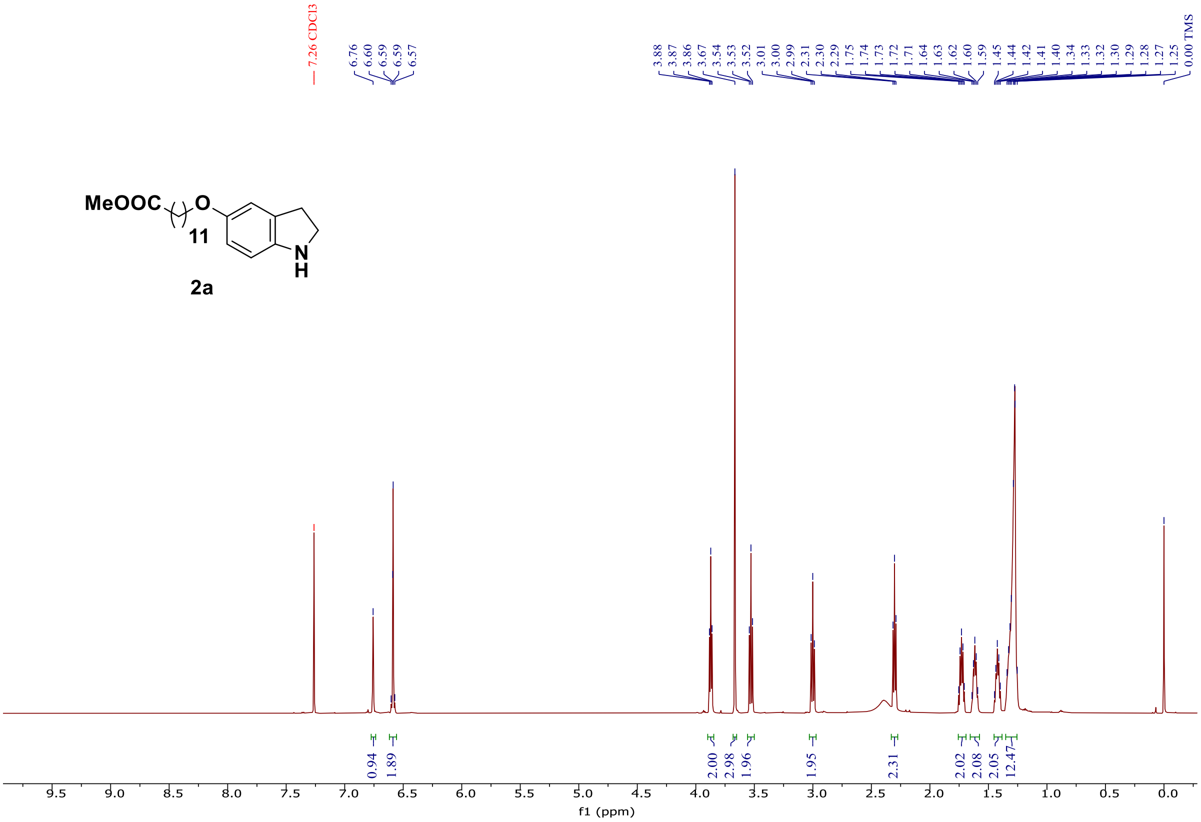

Figure S19. ^{1}H NMR spectrum (600 MHz, 25 °C) of compound **2a** in $CDCl_3$ with 0.03% v/v TMS.

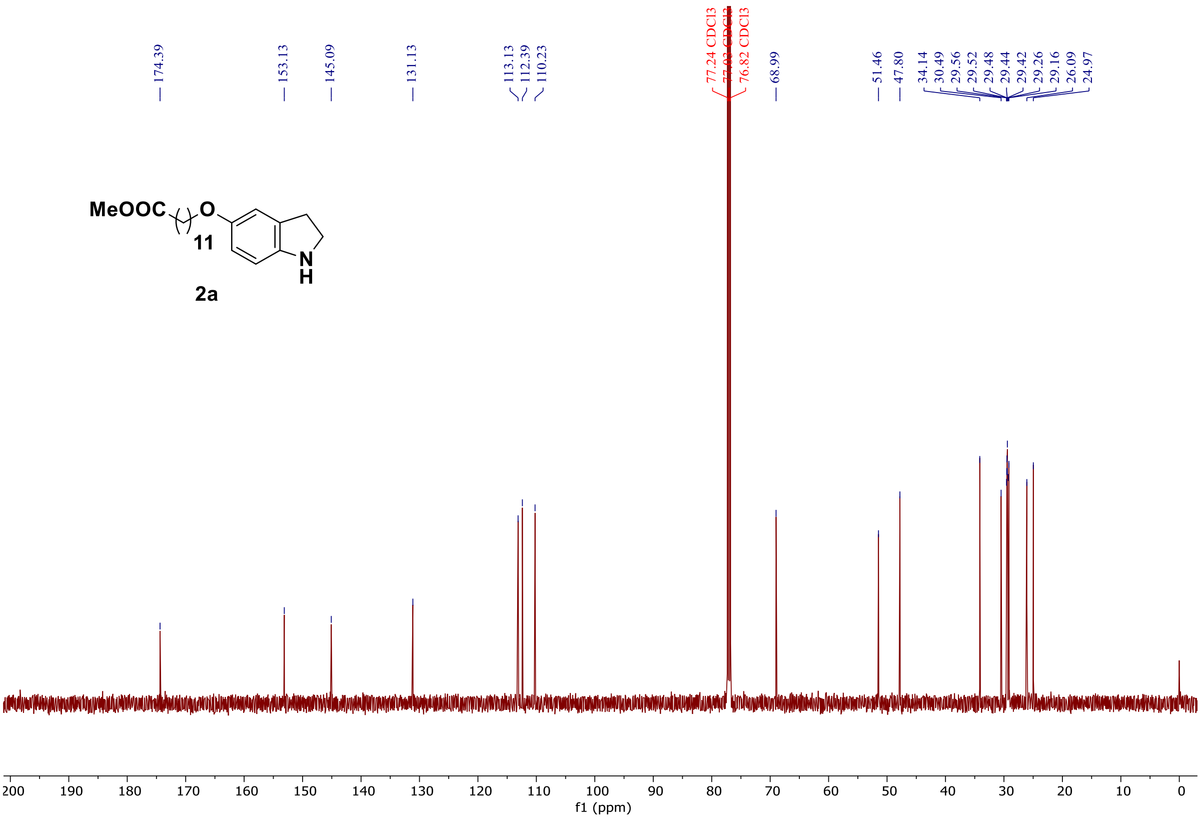

Figure S20. ^{13}C NMR spectrum (151 MHz, 25 °C) of compound **2a** in $CDCl_3$ with 0.03% v/v TMS.

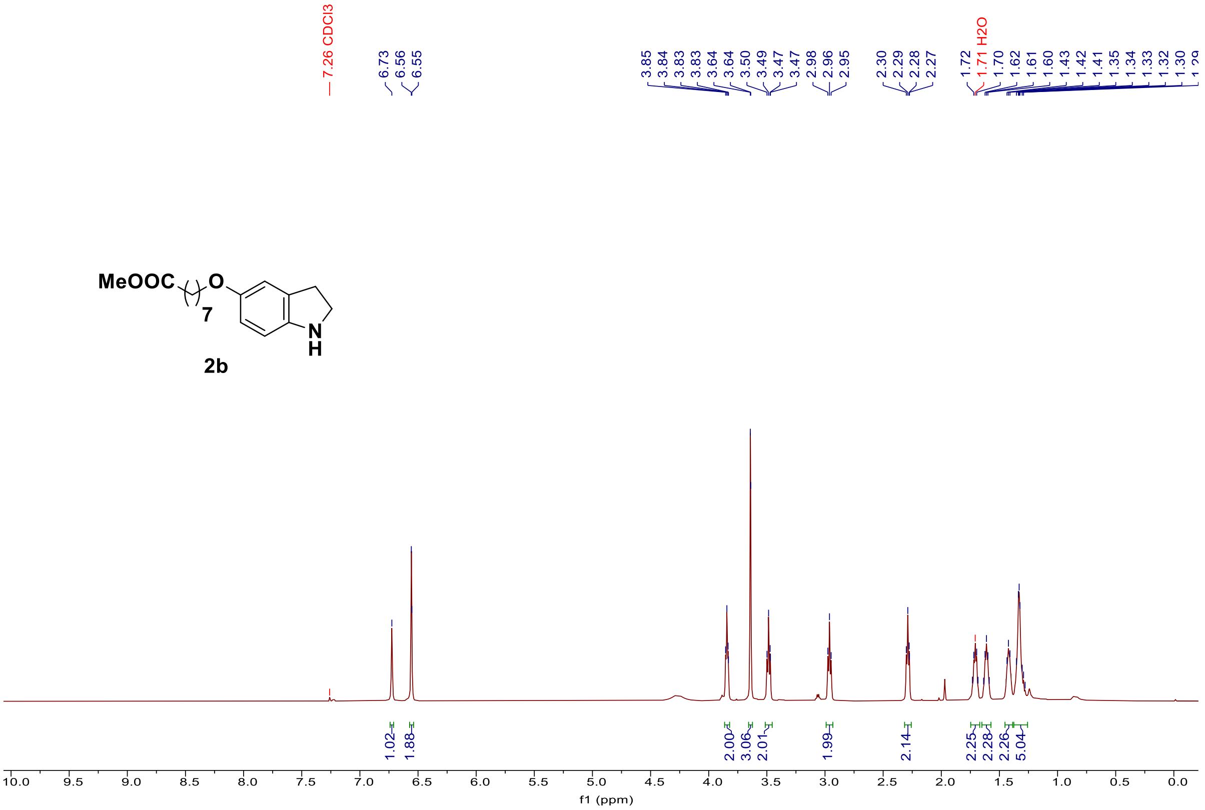

Figure S21. ^{1}H NMR spectrum (600 MHz, 25 °C) of compound **2b** in $CDCl_3$ with 0.03% v/v TMS.

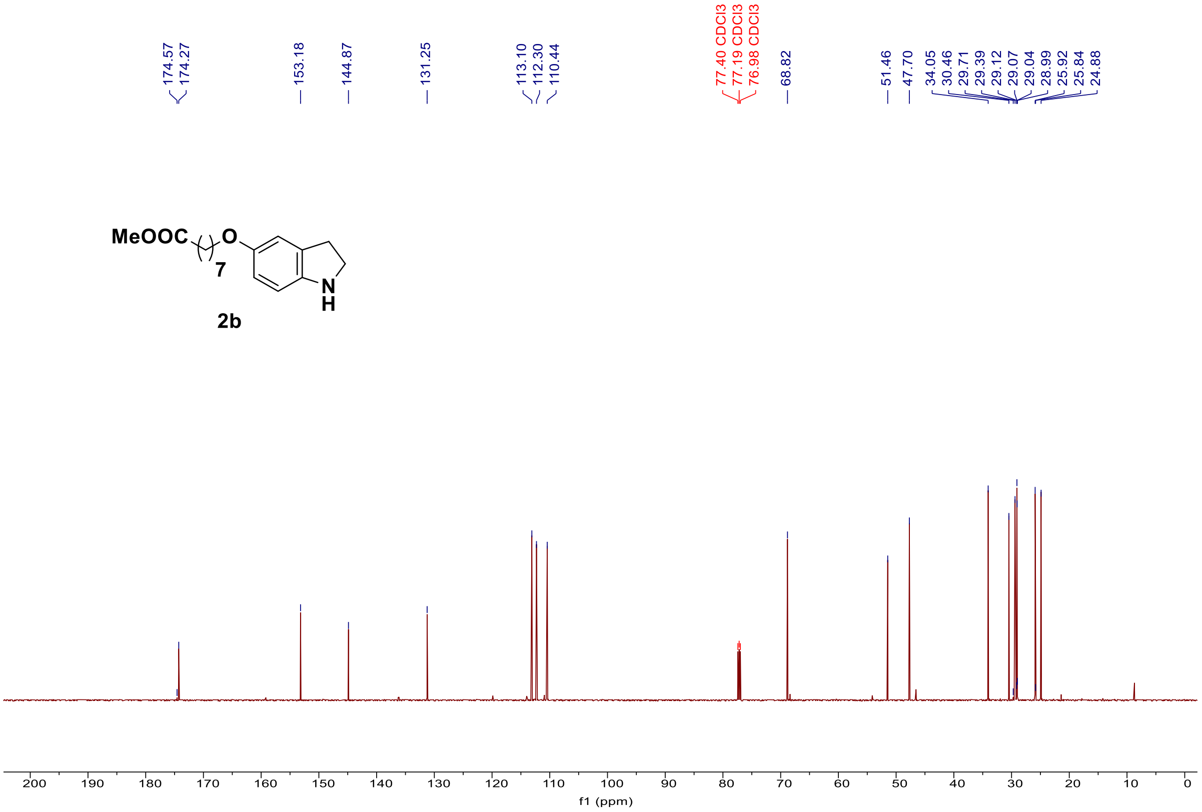

Figure S22. ^{13}C NMR spectrum (151 MHz, 25 °C) of compound **2b** in $CDCl_3$ with 0.03% v/v TMS.

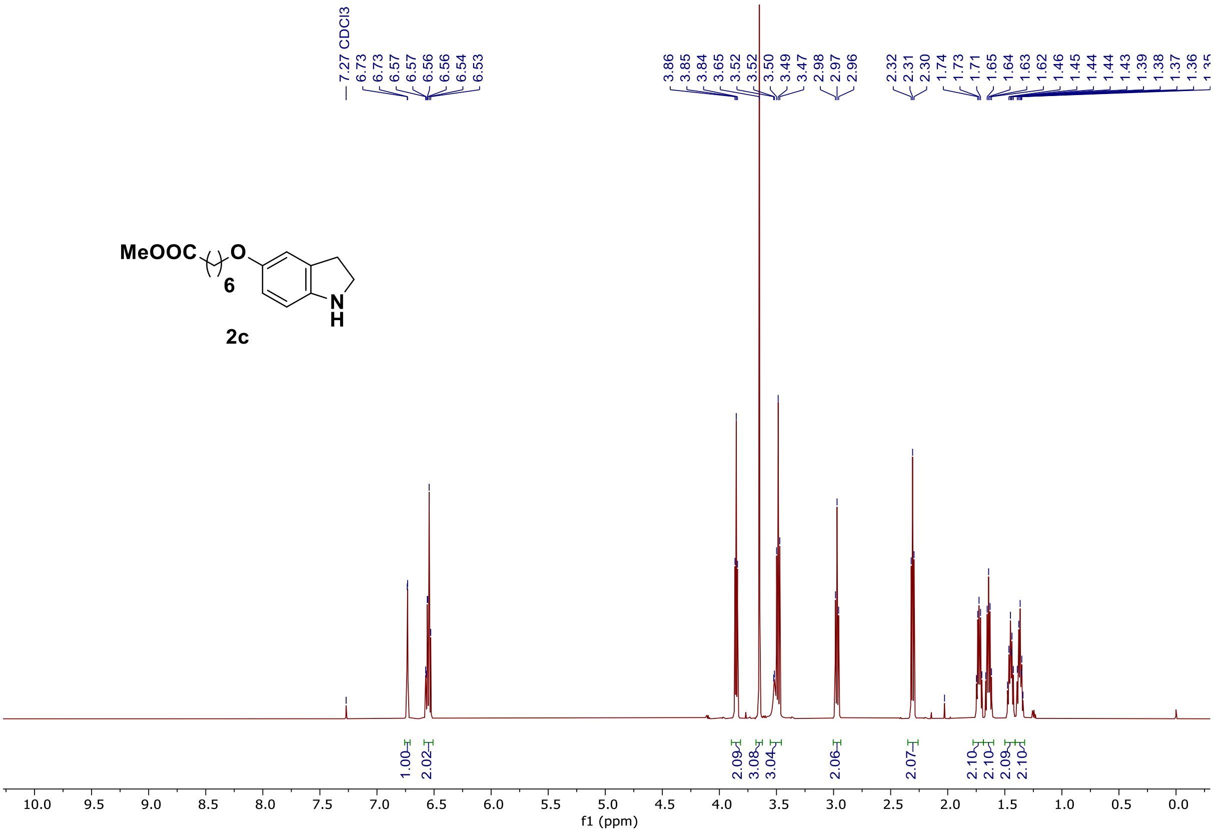

Figure S23. ^{1}H NMR spectrum (600 MHz, 25 °C) of compound **2c** in $CDCl_3$ with 0.03% v/v TMS.

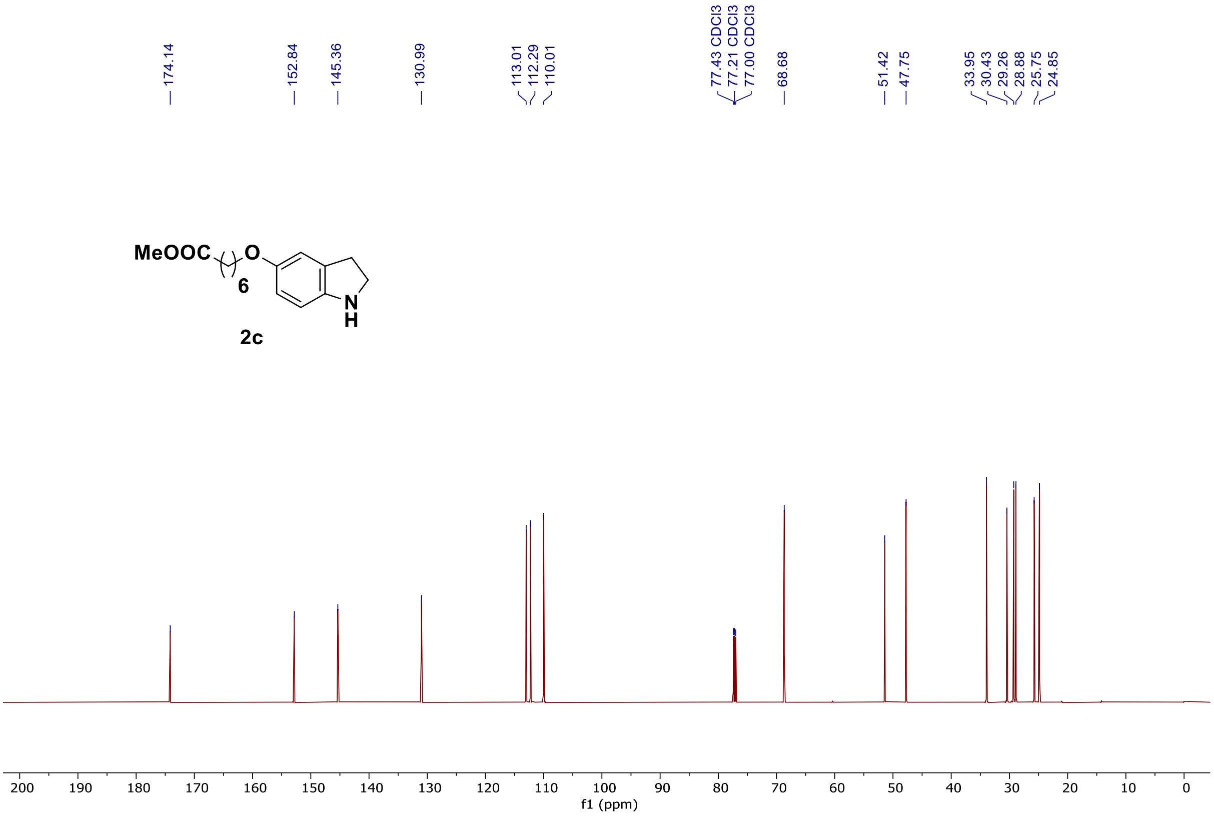

Figure S24. ^{13}C NMR spectrum (151 MHz, 25 °C) of compound **2c** in $CDCl_3$ with 0.03% v/v TMS.

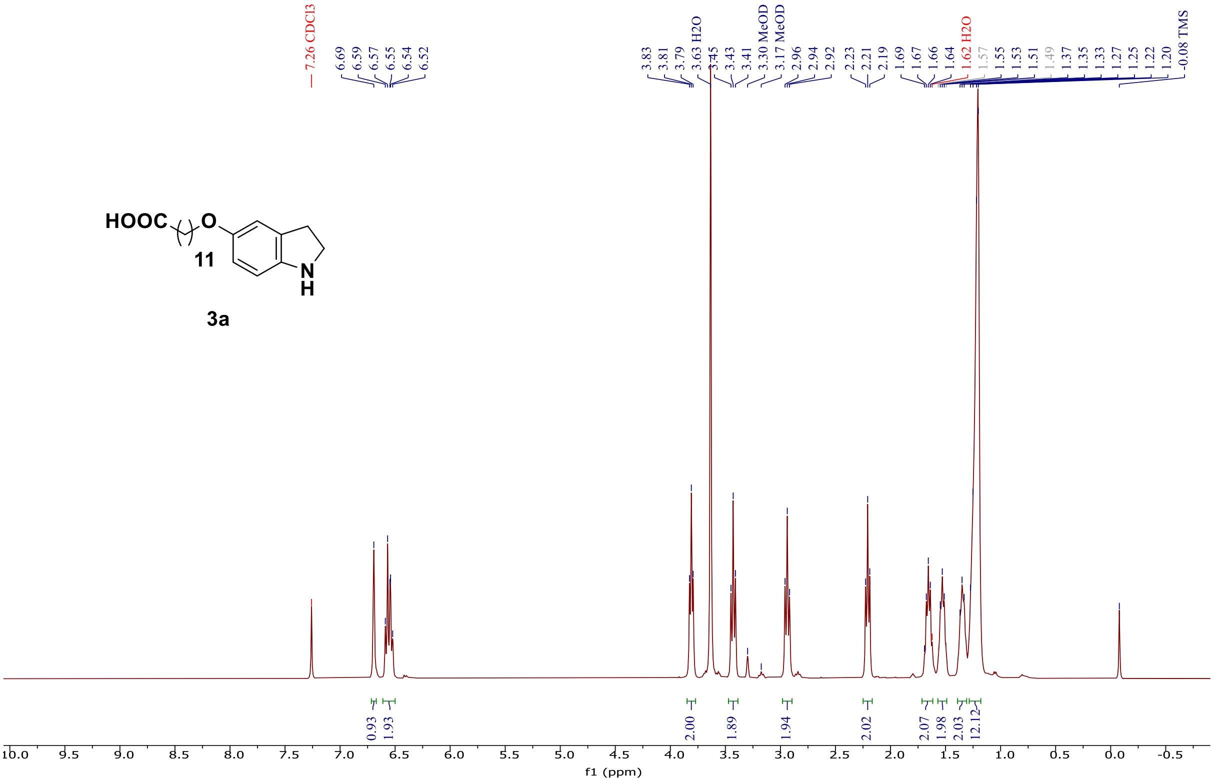

Figure S25. ^{1}H NMR spectrum (600 MHz, 25 °C) of compound **3a** in 9:1 $CDCl_3$ with 0.03% v/v TMS:CD_3OD.

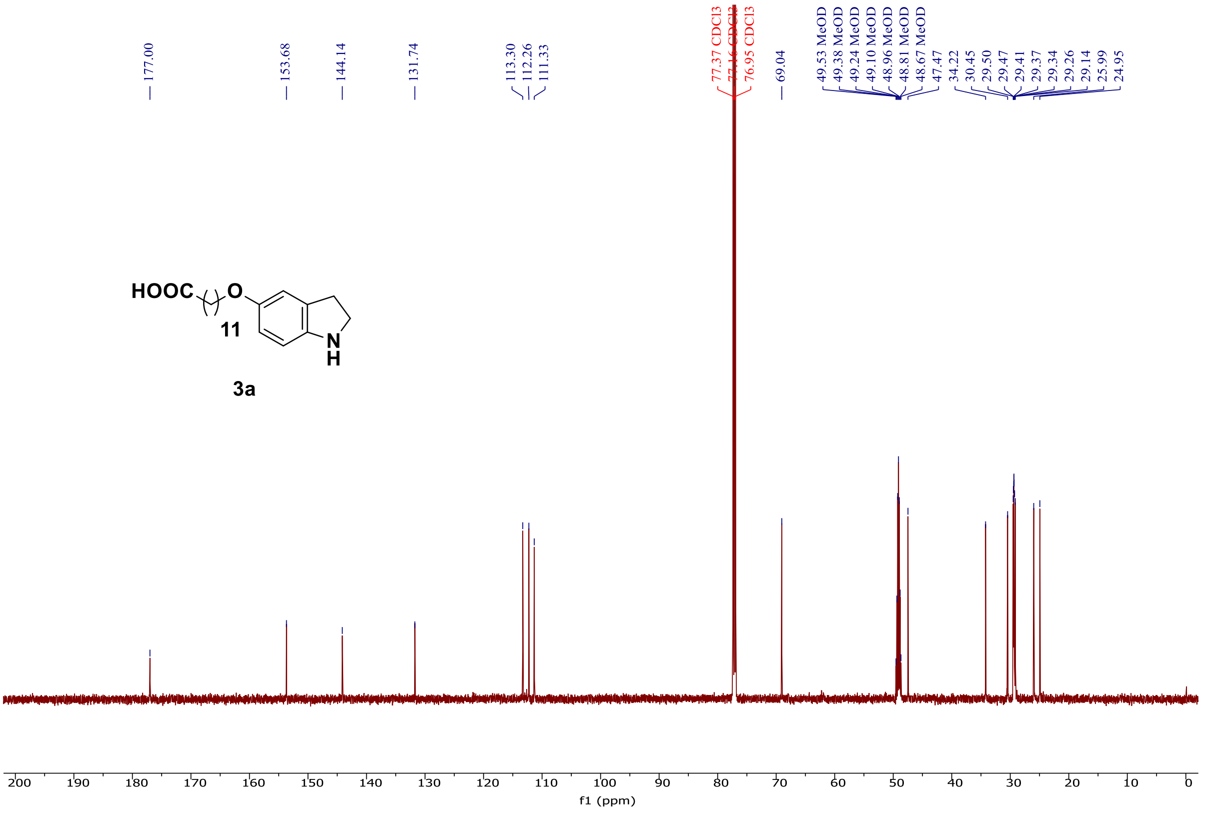

Figure S26. ^{13}C NMR spectrum (151 MHz, 25 °C) of compound **3a** in 9:1 $CDCl_3$ with 0.03% v/v TMS:CD_3OD.

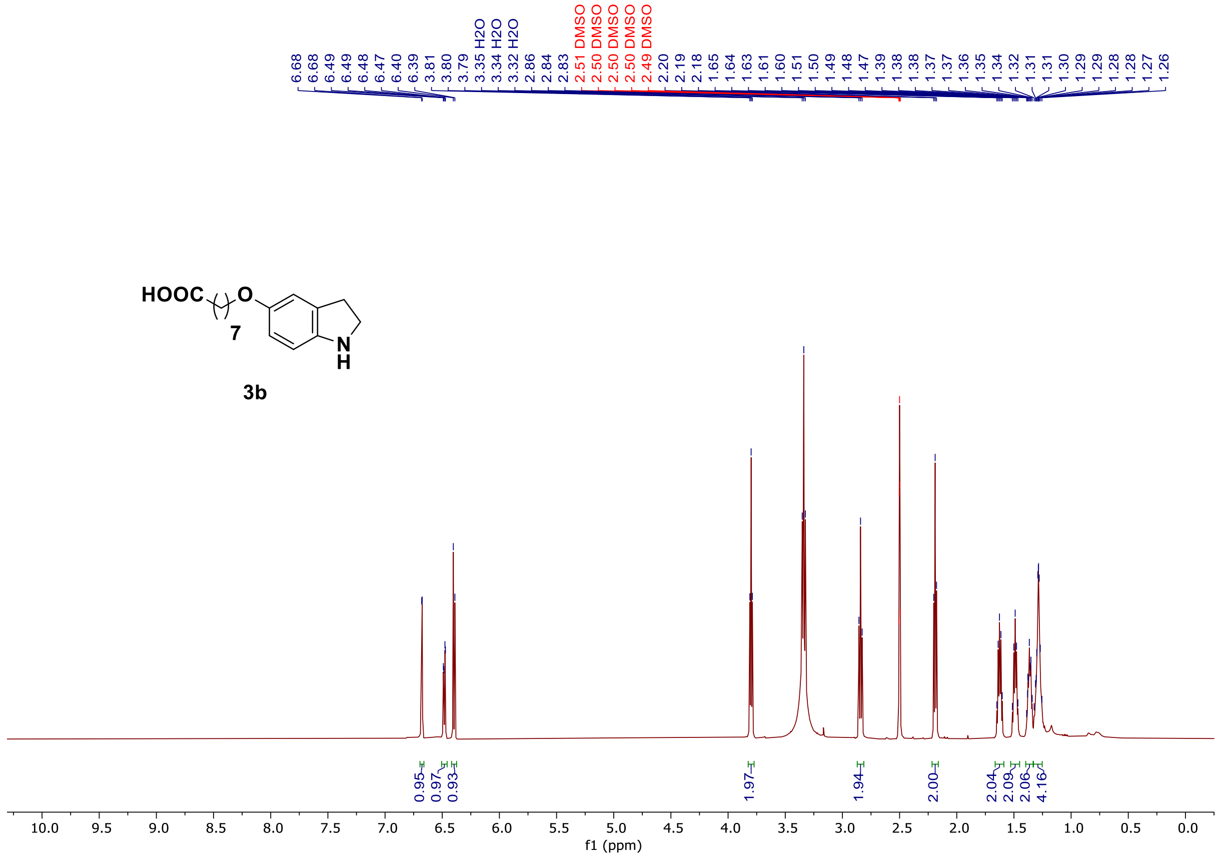

Figure S27. ^{1}H NMR spectrum (600 MHz, 25 °C) of compound **3b** in DMSO-d_6.

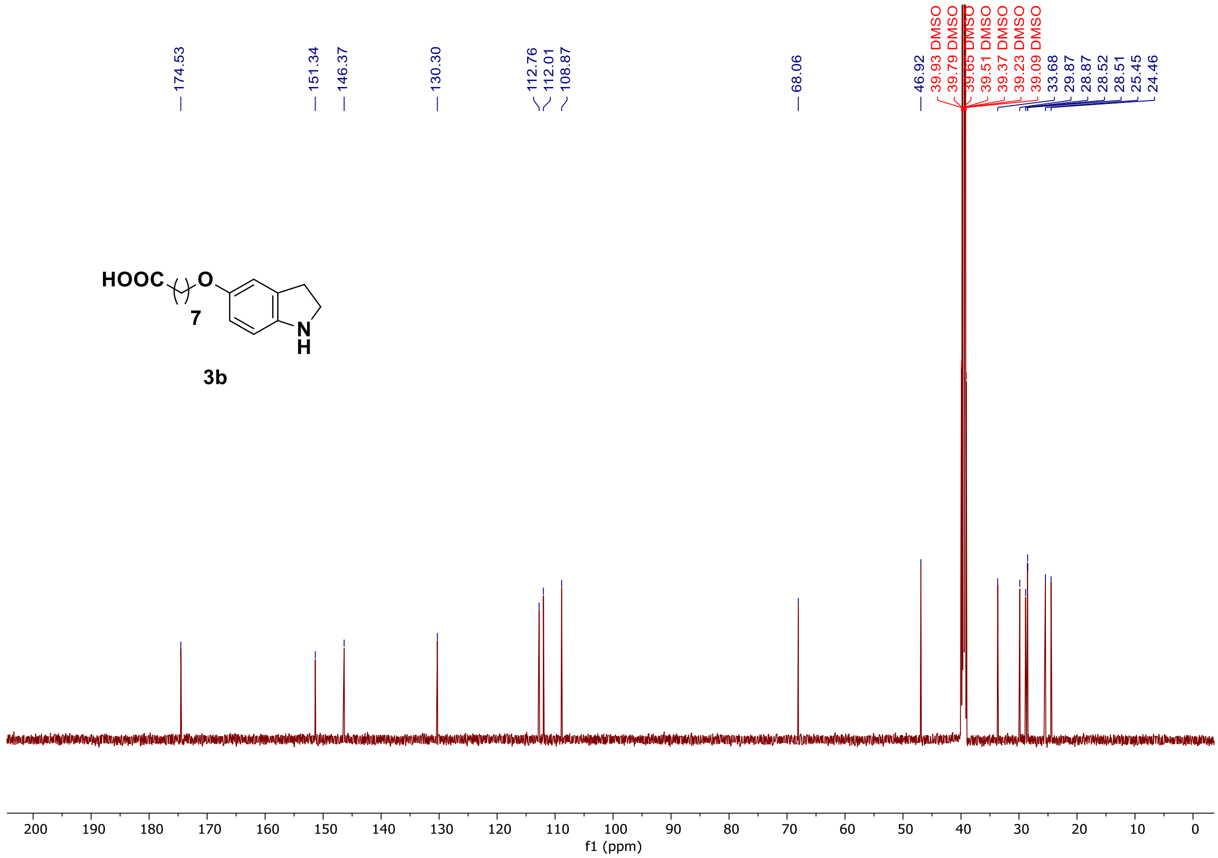

Figure S28. ^{13}C NMR spectrum (151 MHz, 25 °C) of compound **3b** in DMSO-d_6.

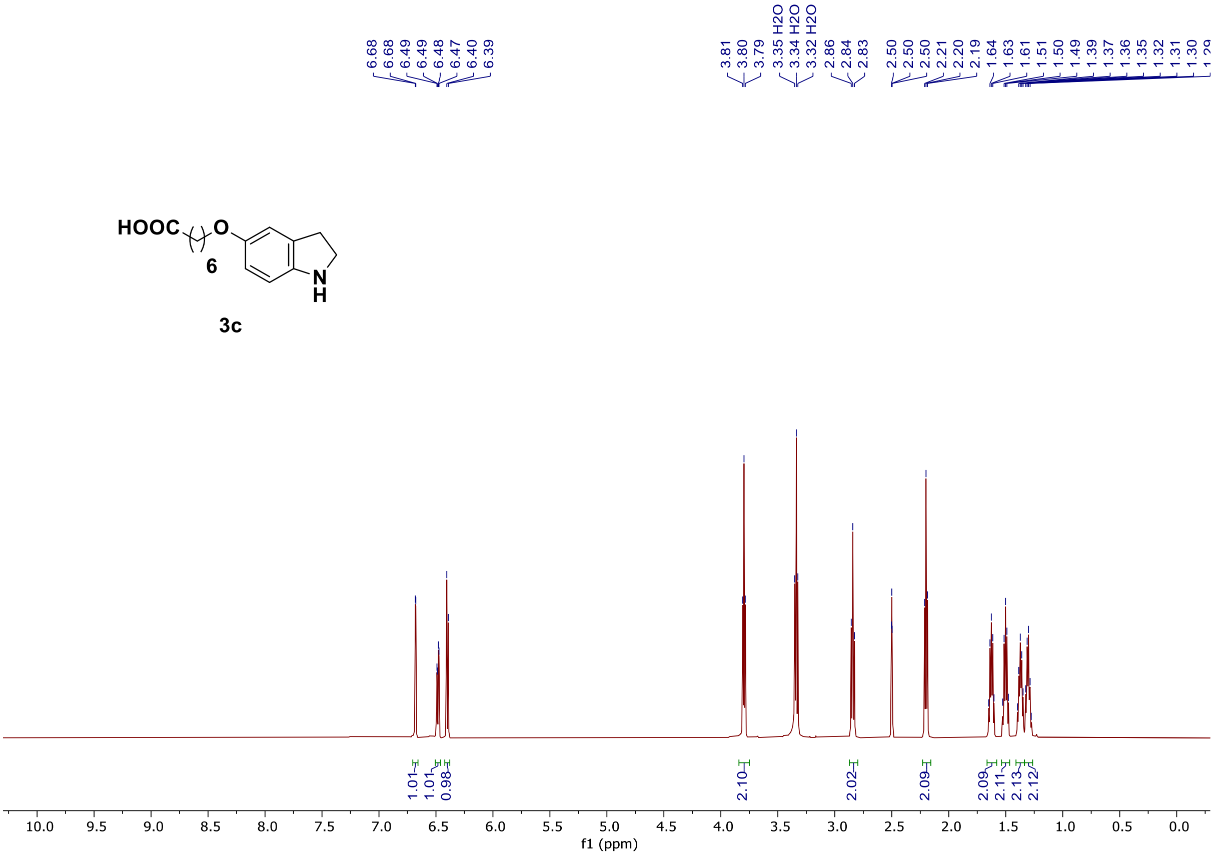

Figure S29. ^{1}H NMR spectrum (600 MHz, 25 °C) of compound **3c** in DMSO-d_6.

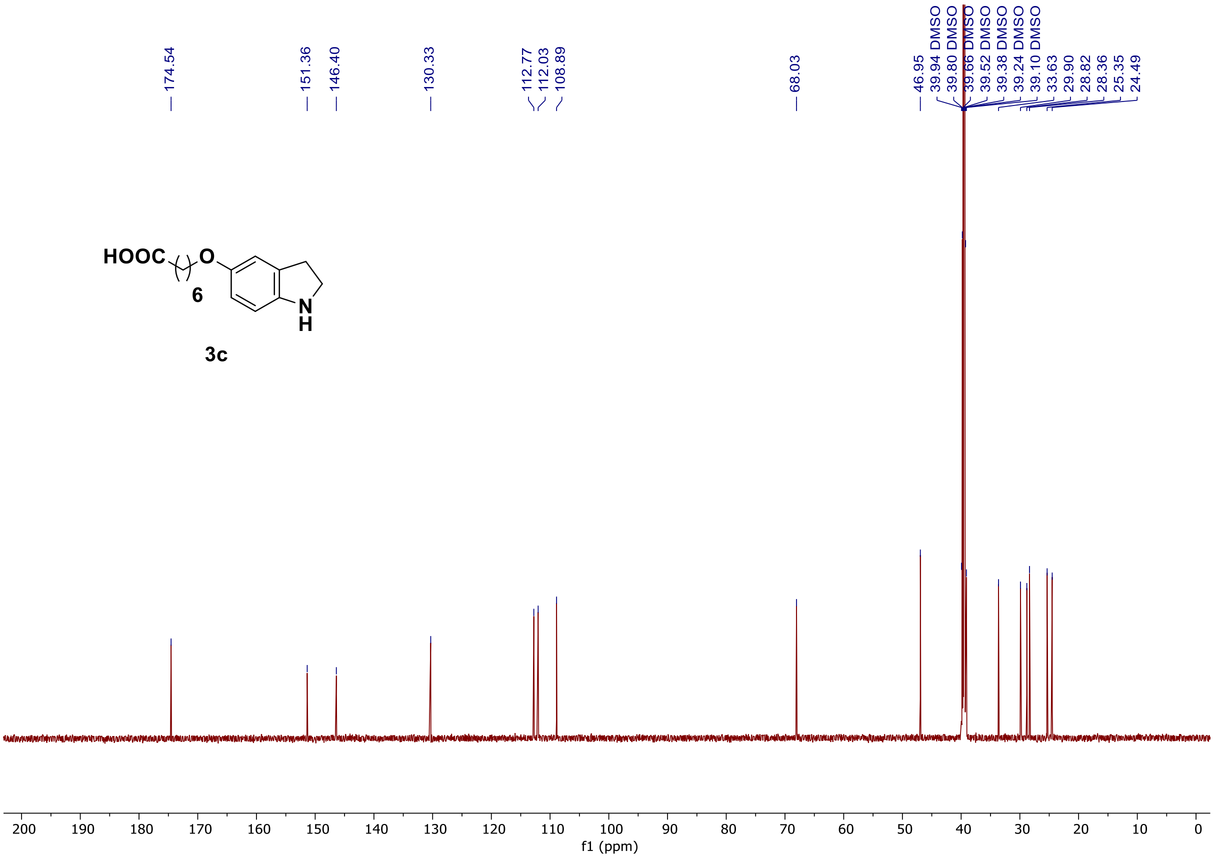

Figure S30. ^{13}C NMR spectrum (151 MHz, 25 °C) of compound **3c** in DMSO-d_6.

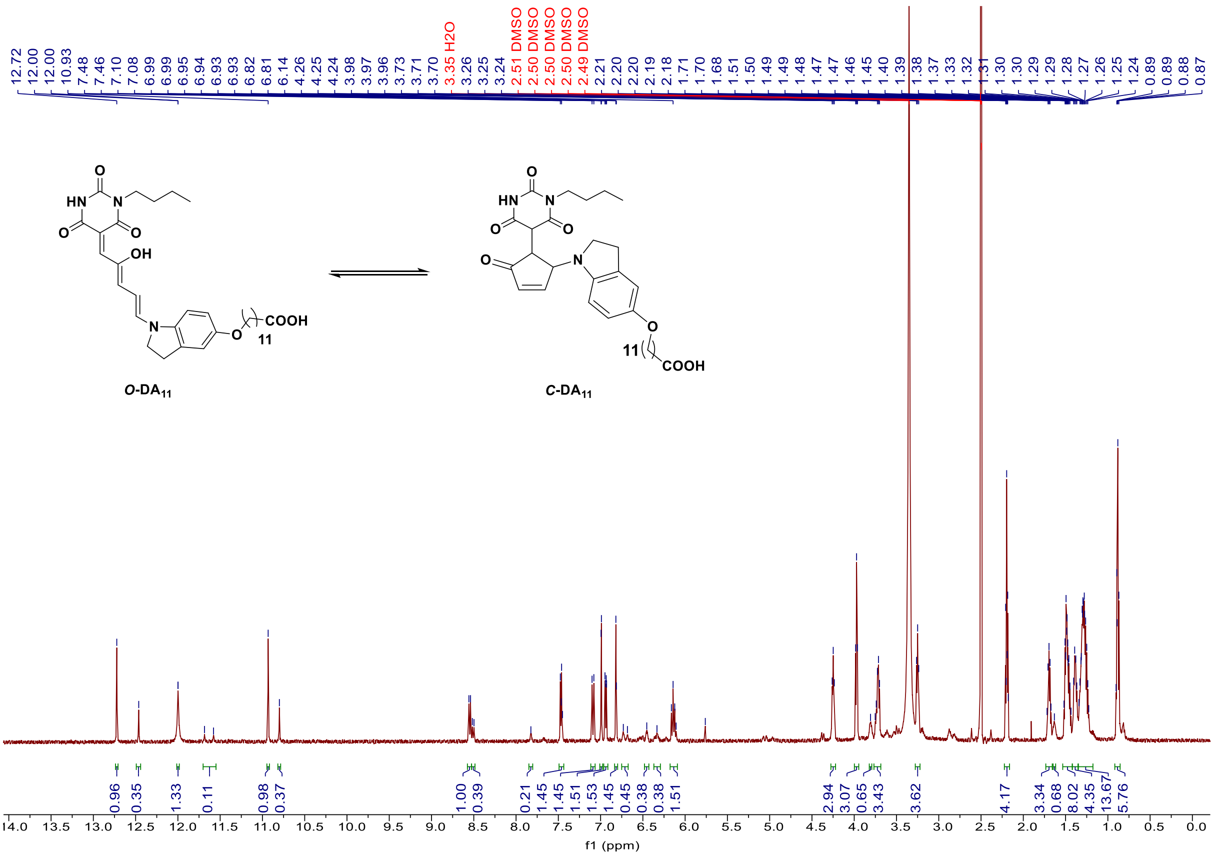

Figure S31. ^{1}H NMR spectrum (600 MHz, 25 °C) of **DA$_{11}$** in DMSO-d_6.

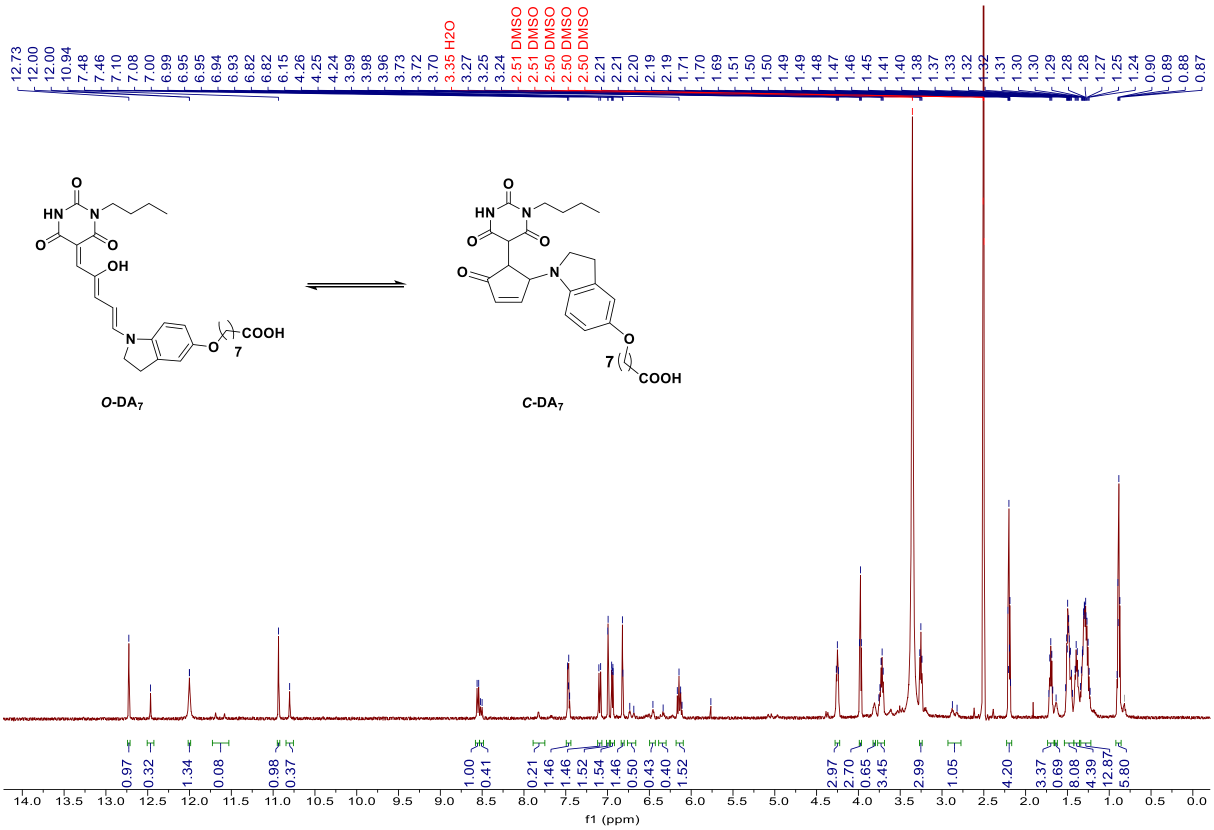

Figure S32. ^{1}H NMR spectrum (600 MHz, 25 °C) of **DA$_{7}$** in DMSO-d_6.

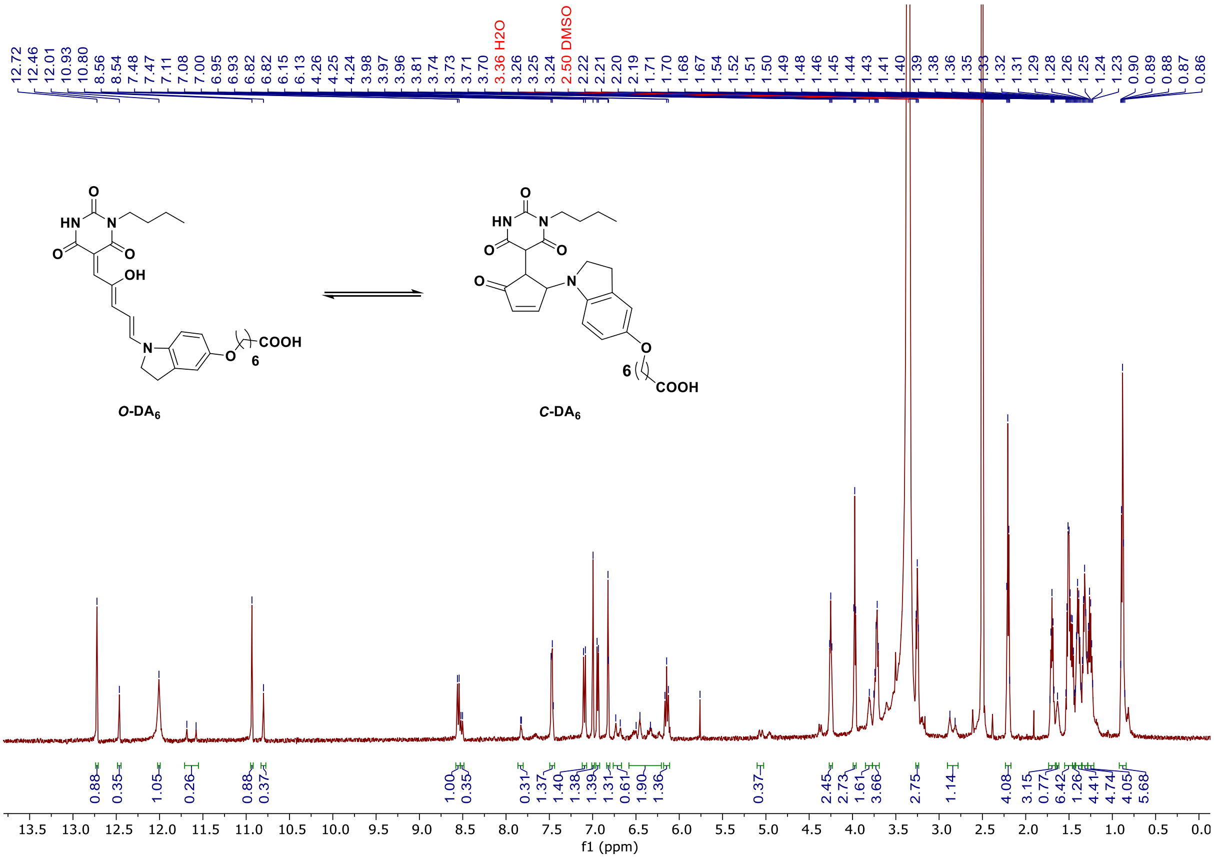

Figure S33. ^{1}H NMR spectrum (600 MHz, 25 °C) of **DA$_6$** in DMSO-d_6

4. References

1. Cheung, L.-H.; Kajitani, T.; Leung, F. K.-C. *J. Colloid Interface Sci.* **2022**, *628*, 984-993.
2. Leung, F. K.-C.; Cui, J.-F.; Hui, T.-W.; Zhou, Z.-Y.; Wong, M.-K. *RSC Adv.* **2014**, *4* (51), 26748-26756.
